# Utility Assessment of Isolated Starch and Extract from Thai Yam (*Dioscorea hispida* Dennst.) for Cosmetic via In Vitro and In Vivo Studies

**DOI:** 10.3390/life15020151

**Published:** 2025-01-22

**Authors:** Suthinee Sangkanu, Jiraporn Khanansuk, Sathianpong Phoopha, Wandee Udomuksorn, Thitiporn Phupan, Jirapa Puntarat, Sucharat Tungsukruthai, Sukanya Dej-adisai

**Affiliations:** 1 Department of Pharmacognosy and Pharmaceutical Botany, Faculty of Pharmaceutical Sciences, Prince of Songkla University, Hat-Yai, Songkhla 90112, Thailand; suthinee.9938@gmail.com (S.S.); jiraporn.kha@psu.ac.th (J.K.); sukanya.d@psu.ac.th (S.D.); 2 Traditional Thai Medical Research and Innovation Center, Faculty of Traditional Thai Medicine, Prince of Songkla University, Hat-Yai, Songkhla 90112, Thailand; sathianpong.p@psu.ac.th (S.P.); 3 Division of Health and Applied Science, Faculty of Science, Prince of Songkla University, Hat-Yai, Songkhla 90112, Thailand; wandee.u@psu.ac.th (W.U.); 6710220007@psu.ac.th (T.P.); jirapa.p@psu.ac.th (J.P.); sucharat.t@psu.ac.th (S.T.)

**Keywords:** *Dioscorea hispida*, dusting powder, cosmetic, facial sleeping mask, tyrosinase, elastase

## Abstract

In Thailand, wild yam, or *Dioscorea hispida* Dennst., is a starchy crop that is usually underutilized in industry. The purpose of this study was to isolate the starch and extract the phytochemical from *D. hispida* and use them in cosmetics. Starch was used instead of talcum, which can cause pulmonary talcosis in dusting powder formulas (DP 1-5). GC-MS was used to identify the bioactive components present in the ethanolic extract of *D. hispida*. The main compounds were identified as 9,12-octadecadienoic acid (Z,Z)- (6.51%), stigmasta-5,22-dien-3-ol, (3.beta.,22E)- (6.41%), linoleic acid ethyl ester (5.72%), (Z,Z)-9,12-octadeca-dienoic acid, 2,3-dihydroxy-propyl (3.89%), and campesterol (3.40%). Then, the extract was used as an ingredient in facial sleeping mask gel formulas (SM 1–SM 5). Stability tests, physical characteristics, enzyme inhibitions, and sensitization dermal toxicity tests were used to evaluate the DP and SM formulations. The results showed that the fresh tubers of *D. hispida* showed a 12.5% *w*/*w* starch content. The findings demonstrated that starch powder had a restricted size distribution, ranging from 2 to 4 μm, and a smooth surface that was polygonal. Following stability testing, the color, odor, size, and flowability of all DP formulations did not significantly differ. The SEM investigation revealed that DP particles were homogenous. For the sensitization dermal toxicity test, DP denoted no erythema or skin irritation in the guinea pigs. After stability testing, the colors of the SM formulas were deeper, and their viscosity slightly increased. The pH did not significantly change. After the stability test, SM formulas that contained *Glycyrrhiza glabra* and *D. hispida* extracts exhibited stable tyrosinase and elastase inhibitory activities, respectively. In the sensitization dermal toxicity test, guinea pigs showed skin irritation at level 2 (not severe) from SM, indicating that redness developed. All of these findings indicate that *D. hispida* is a plant that has potential for use in the cosmetics industry. Furthermore, *D. hispida* starch can be made into a beauty dusting powder, and more research should be conducted to develop an effective remedy for patients or those with skin problems.

## 1. Introduction

Plants play the most important role in human food. In addition to the essential elements of protein and carbohydrates, plant-based diets also include a variety of secondary metabolites, often known as natural products. Plant secondary metabolites are not necessary for the growth of plants, but they are necessary for plants to interact with the external environment and develop in response to stress, such as antimicrobial properties that protect the plant from infections [1], ultraviolet protection [2], and antioxidants [3]. Furthermore, because of secondary metabolite characteristics, they have been used in a wide range of other applications as pigments, medicine, cosmetics, and more [4].

Cosmetics are created to be applied to the human body in order to change appearance, promote attractiveness, clean, or beautify. These days, there is an increasing demand for natural items to be used as cosmetic components due to the fact that formulations utilizing phytochemicals from various plant sources show benefits without creating negative effects [5]. *Dioscorea* spp. is a genus of the family Dioscoreaceae in the order Dioscoreales. This genus comprises over 600 species [6]. In Thailand, three varieties, *D. alata* L., *D. esculenta* (Lour.) Burkill, and *D. hispida* Dennst., are grown, but there is limited commercial yam farming of these three varieties [7]. *Dioscorea* generate starchy tubers that are important for nutrition, culture, and the economy. Its tuber contains a large number of nutritional and functional ingredients, including starch, fiber, protein, polysaccharides, steroid saponins, dioscorine, allantoin, flavonoids, polyphenols, and other active compounds [8,9]. These ingredients have the potential to be therapeutically useful and have health benefits like antimicrobial, antioxidant, anti-inflammatory, anticancer, anti-diabetic, anti-obesity, and anti-hypercholesterolemic activities [10]. *Dioscorea* tubers have approximately 76% starch, 10% protein, 0.22% fat, 1.2% crude fiber, and a few minerals (approximately 135 mg/kg Ca and 8.3 mg/g K) on a dry weight basis [11]. Previous studies of *D. hispida* starch have demonstrated that starch has a moisture content of 9.45%, starch content of 37.62% [12], and oil absorption of 16.85% [13]. The starch granules were discovered under a scanning electron microscope (SEM) to have polyhedral forms and range in size from 1.3 µm to 4.3 µm. When increasing the temperature ranging from 65 °C to 95 °C, *D. hispida* starch showed an increase in swelling and solubility [14].

Dusting powders (DPs) are split into small fine powders that are applied to intact skin. DPs are used for various purposes, such as treating certain skin disorders, reducing chafing, absorbing moisture, reducing irritation, and local use. The following qualities are desirable in DPs: good absorption, compatibility with skin secretions, free flowability, easy spreadability, non-irritability, and non-grittiness [15]. Natural starch is widely used in the food, pharmaceutical, and cosmetic industries and is naturally produced by plants, animals, sea algae, and microbes. Among these sources, plants have been considered the best sources of starch because of their extensive availability, inexpensiveness, simple extraction, and non-toxicity [16]. The use of natural starch in cosmetics has attracted a lot of attention due to its ability to enhance texture, dispersion stability, biodegradability, skin and mucus biocompatibility, and sensory abilities [17]. Rincón and colleagues reported that guapo (*Myrosma cannifolia*) starch has been substituted for talc in the creation of face powders [18]. Additionally, sago (*Metroxylon sagu* Rottb.) starch has been used in body dusting powder formulas. Perfumed and cooling body powders with sago starch had satisfaction ratings that were comparable to those of commercial products [19].

The skin is recognized as the largest organ and functions as a protective barrier against the infection of microbes in the body. Consequently, maintaining skin health is a crucial component of overall personal well-being. Additionally, skin conditions can significantly influence psychosocial interactions and communication among individuals [20]. Within the market, the category of “facial” treatments is the most prevalent, encompassing a variety of products including creams, lotions, peels, and masks [21]. Facial masks are widely available products that are simple to use and provide immediate benefits for the skin. There are various types of face masks, including gel, emulsion, sheet, and paste [20]. These masks are formulated with bioactive components that operate via various mechanisms to impart rejuvenating effects. Such ingredients may include moisturizers, exfoliants, brightening agents, herbal extracts, and an array of vitamins, proteins, and minerals. In the cosmetic field, there have been studies and applications of *D. hispida* extract as an ingredient in rejuvenating facial soap bars [22]. Moreover, *D. hispida* extract is used to lessen wrinkles and pigmentation. *D. hispida* tuber extract was discovered to have strong hypoglycemic, anticancer, and antioxidant qualities [23]. Due to the synergistic action of several biomolecules, the phenolic compounds included in *D. hispida* tubers have the ability to act as antioxidants and prevent wrinkles [22].

Therefore, instead of using talcum as the primary ingredient in the DP formula, the starch components from the *D. hispida* tubers were isolated for this investigation. Furthermore, the bioactive ingredients from *D. hispida* tubers were isolated and added to the sleeping mask formula for the face in order to prevent wrinkles. Finally, we assessed the sensitization dermal toxicity of the formulations.

## 2. Materials and Methods

### 2.1. Preparation of D. hispida Starch

*D. hispida* used in this study was obtained from Lansaka District, Nakhon Si Thammarat Province, Thailand. Samples were collected in April 2022. *D. hispida* tubers were cleaned, peeled, and cut into small pieces. Prior to the starch isolation, the samples were cleaned under running water for 24 h to eliminate the alkaloid dioscorine. Then, the samples were blended at room temperature using a ratio of 1:1.5 *D. hispida* tubers to water, and the starch suspension was then left overnight for sedimentation in a box filled with ice for 2 days. The settled starch layer was then aliquoted to the general test of alkaloids for dioscorine determination, and steroid saponin was performed using the Liebermann–Burchard test. After that, the settled starch layer was centrifuged (Kubota, 5922, Tokyo, Japan) at 4 °C and a speed of 4000 rpm for 15 min, and the upper liquid was discarded. The settled starch layer was washed twice with distilled water. In the final stage, the starch was dried at 55 °C for 2 days in the oven. The size of starch was reduced using a mortar and pestle and sieved using an Auto sieve (Retsch, AS200, Haan, Germany) with pore sizes of 355, 250, and 180 µm.

### 2.2. Extraction of Pectin from Dragon Fruit Peel

About 10 kg of dragon fruit (*Hylocereus undatus* (Haw.) Britton & Rose) peel were cut into small pieces and boiled for 30 min in deionized water. After that, the liquid portion was filtered through cheesecloth and allowed to cool in the refrigerator for an hour. Cool ethanol was used in a 1:1 (*v*/*v*) ratio to precipitate pectin. Then, we used two layers of cheesecloth to filter the pectin. Following that, the pectin was transferred to a tray, and the ethanol was left to evaporate for 2 to 3 h before it was freeze-dried (Freeze Dryers, Labconco, KS, USA).

### 2.3. Preparation of D. hispida Extract

First, 7.5 kg of *D. hispida* tubers were cut into small pieces, cleaned a couple of times with water, and then the material was extracted using the reflux method for 5 h with 80% ethanol as the solvent. To obtain the extract, the solvent was then drained, filtered through filter paper, and freeze-dried. The extract was kept at −20 °C in an airtight container.

### 2.4. Glycyrrhiza glabra Extraction

The extract of *G. glabra* was collected by the maceration process. It was carried out by soaking 50 g of *G. glabra* stem powder in 100 mL of 80% ethanol with a shaker at 90 rpm and 25 °C for 72 h. After that, the extract was filtered through Whatman No. 1 filter paper. The solvents were separated from the extract with the aid of a rotary evaporator (Hei-Vap Value, Heidolph, Schwabach, Germany) at 65 °C.

### 2.5. Gas Chromatography–Mass Spectrometry Analysis (GC–MS Analysis)

The sample was submitted to the Office of Scientific Instrument and Testing (OSIT) at Prince of Songkla University for GC-MS analysis. The sample was examined using a 7820A gas chromatograph connected to a 5975C network mass spectrometer (GC–MS) (Agilent Technologies, Waldbronn, Germany). The separation column was an Agilent Technologies HP-5MS 5% Phenyl Methyl Silox column (30 m × 250 µm × 0.25 µm). After being set at 80 °C and maintained for three minutes, the column temperature was raised at a rate of 5 °C per minute to 280 °C and then maintained for five minutes. It required 48 min to run in total. There was a 10:1 split ratio and a 1 µL injection volume. The carrier gas, helium, was employed at a flow rate of 1 mL/min. Using electron ionization (EI) at 70 eV and full-scan acquisition mode, MS detection was carried out in the *m*/*z* range of 35–350. Only chemicals that accounted for more than 1% of all detected compounds and had a percent matching factor of greater than 90% from the database were taken into consideration by the authors.

### 2.6. Preparation of Dusting Powder (DP) from D. hispida Starch

The powder of *D. hispida* starch was sieved using mesh number 80 (180 µm) to ensure consistency and fineness of particle size. We weighed the *D. hispida* starch powder amount that was provided, as well as the components listed in Table 1. Then, using the geometric dilution approach, powder granules were mixed with zinc oxide, magnesium stearate, kaolin, and phenoxyethanol (all standard chemicals were purchased from Sigma-Aldrich, Steinheim, Germany). The combined powder was mixed with *G. glabra* extract, and then a coloring agent was added. The combined powder was allowed to dry at 50 °C for 60 min before being sieved through mesh number 100 (150 µm). DP was packed into a sealed container.

### 2.7. Preparation of Sleeping Mask (SM) from Pectin of Dragon Fruit Peels

We experimentally used pectin from dragon fruit peels in gel formulations instead of Carbopol 934. The ingredients were weighed in accordance with Table 2. Dragon fruit peel pectin was first ground into a fine powder in a mortar and transferred to a beaker. Then, the glycerin (Vidhyasom Co., Ltd., Bangkok, Thailand), propylene glycol (C.P. Drug Center Co., Ltd., Bangkok, Thailand), and 15 mL of water were added. After thorough stirring, they were maintained until the gel swelled and turned transparent. Next, sodium benzoate (Sigma-Aldrich, Steinheim, Germany), *G. glabra*, and *D. hispida* extract solution were added. Finally, the remaining water was added until the volume reached 25 mL and stirred to create a clear gel. A sealed container was used to hold the SMs in a refrigerator.

### 2.8. Accelerated Stability Testing

For the stability study of the DP and SM formulations, the freeze–thaw cycle was modified and used [24]. The freeze–thaw cycle was prepared as follows: the sample was placed in the stability incubator at −5 ± 2.0 °C for 24 h and then thawed at 45 ± 2.0 °C for 24 h; this process was a cycle. Six freeze–thaw cycles were performed (12 days). Characteristics such as color, odor, texture, average particle size, flowability, viscosity, pH, and enzyme activities were assessed on the first and twelfth days.

### 2.9. Particle Size

The particle size of the developed DPs was determined using optical microscopy and an OLYMPUS EP50 microscope digital camera (Tokyo, Japan). The size was randomly measured using the EPview 1.2 application. The average value was calculated and recorded.

### 2.10. Test for Flowability

According to the angle of repose, the funnel method was used to assess the powder’s particle flow. We weighed 15 g of powder and then transferred the material into a 5.7 cm diameter disc using a funnel with a 0.6 cm diameter. Once the material had gone down the funnel to its fullest extent, we measured its height in the disc and used the calculation to determine the angle of repose, or θ (1). The classification of angle repose by Carr is shown in Table 3 [25].

Tan θ = h/r (1)

where θ is the angle of repose, r is the radius of the conical pile, and h is the height of the conical pile.

### 2.11. Scanning Electron Microscopy (SEM)

The surface morphology of the starch granules in DPs was assessed using a scanning electron microscope (Thermo Fisher Scientific Quanta 400, Waltham, MA, USA). The dried powder sample was put on a metal stub using double-sided sticky tape and vacuum-coated with gold powder in high-vacuum conditions to produce an electron beam. The image was recorded at an acceleration potential of 20 kV.

### 2.12. Anti-Tyrosinase Activity

The anti-tyrosinase activity of each sample was performed using L-DOPA as a substrate [26]. The reaction mixture (200 µL) contained 20 µL of mushroom tyrosinase (203 unit/mL), 20 µL of the sample, and 160 µL of 2.5 mM L-DOPA in 20 mM phosphate buffer. We measured the absorbance at 492 nm with a microplate reader (time 0). After incubation at 37 °C for 20 min, the tyrosinase activity was monitored at 492 nm for dopachrome formation (time 20) using a Thermo LUX PH5404 Microplate Reader (Watthana, Bangkok). Kojic acid and water extract of *Artocarpus lacucha* were used as positive controls. The percentage of tyrosinase inhibition was calculated using Equation (2).

% tyrosinase inhibition = [(A − B) − (C − D)] × 100/(A − B)(2)

where A, B, C, and D are the differences in light absorption at a wavelength of 492 nm before and after incubation.

### 2.13. Anti-Elastase Activity

An anti-elastase assay [27] was performed in 0.2 mM Tris-HCL buffer (pH 8.0). Porcine pancreatic elastase was dissolved in cool sterile water to make a 0.3 unit/mL. The substrate N-Succinyl-Ala-AlaAla-*p*-nitroanilide (AAAPVN) was dissolved in a buffer at a concentration of 1.6 mM. The sample (25 µL) was incubated with the enzyme (25 µL) in a buffer (50 µL) for 15 min at room temperature; after that, the substrate (25 µL) was added to begin the reaction. EGCG (2 mg/mL) and 20% DMSO were used as positive and negative controls, respectively. Absorbance values of 410 nm were measured immediately following the addition of the substrate and then continuously for 20 min using a Thermo LUX PH5404 Microplate Reader. The percentage of elastase inhibition was calculated using Equation (3).

%Inhibition = [(V control − V sample)/V control] × 100(3)

### 2.14. Sensitization Dermal Toxicity Test

Male guinea pigs (Dunkin-Hartley) (10–12 weeks old and weighing 350–450 g at receipt) from the National Laboratory Animal Center at Mahidol University were housed in the Southern Laboratory Animal Center at Prince of Songkla University. The animals were kept under conditions of a temperature of 25 ± 2 °C, relative humidity of 50 ± 5%, a photoperiod 12:12 h of a light–dark cycle, and food and water were provided ad libitum. They were allowed 7 days to acclimate to their housing environment before testing. All animal protocols in this study were approved by the Institute Animal Care and Use Committee of Prince of Songkla University under code MHESI 68014/1836.

For skin preparation, the hairs on the backs of animals were carefully shaved 24 h before application. Then, all animals were weighed, and skin areas were photographed before the experiment. There were seven treatments, namely 0.9% NSS (control), powder of *D. hispida*, DP 2, SM 1, SM 2, SM 3, and SM 5, that were chosen for testing. Each treatment was applied to the test areas (about 4 × 2 cm^2^), and they were observed for erythema and edema at 6, 21, 30, and 54 h after the removal of each treatment and given the scores according to Table 4. After the treatments, rechallenges were performed for 4 cycles, including the first time. The degree of skin irritation for each treatment was calculated using the formula below (4) and classified into 5 levels, including level 1 (score 0–8 = no irritated reaction), level 2 (score 9–28 = not severe), level 3 (score 29–67 = moderate), level 4 (score 68–80 = severe), and level 5 (score 81–100 = very severe).

(sum of erythema/edema of the skin × 100)/(number of test animals × number of observation × highest score (4) × skin response*)(4)

where the skin response* is 1 if there is an occurrence of erythema or edema of the skin and 2 if there are occurrences both of irritation.

## 3. Results

### 3.1. Identification of D. hispida Ethanol Extract Using GC-MS

Cosmetic substances used in sleeping masks and dusting powders are derived from *D. hispida*. Therefore, the current work was conducted to use GC-MS to identify the bioactive components present in the ethanolic extract of *D. hispida*. To ensure accurate metabolite determination, the criterion was that the percent match factor of tested metabolites showed more than 90% prediction from the MS database. The results indicated that the extract of *D. hispida* included all 18 major and minor components, as shown in Table 5. The main compounds identified based on relative contents were 9,12-octadecadienoic acid (Z,Z)- (6.51%), stigmasta-5,22-dien-3-ol, (3.beta.,22E)- (6.41%), linoleic acid ethyl ester (5.72%), (Z,Z)-9,12-octadeca-dienoic acid, 2,3-dihydroxy-propyl (3.89%), and campesterol (3.40%). All GC-MS data are provided in the Supplementary Figure S1.

### 3.2. Enzyme Inhibitory Activity of G. glabra and D. hispida Extracts

Two plant species, *G. glabra* and *D. hispida,* were the primary plant extracts used in this investigation. When *G. glabra* extract was tested for tyrosinase activity, it inhibited 82.56 ± 0.89% of the enzyme at a concentration of 20 µg/mL, with an IC_50_ value of 3.97 µg/mL. The extract from *D. hispida* exhibited anti-elastase activity by 80.71 ± 1.38% at a dosage of 2 mg/mL, with an IC_50_ value of 15.13 µg/mL.

### 3.3. Starch Isolation and Dusting Powder (DP) Formulation

**Figure 1:**
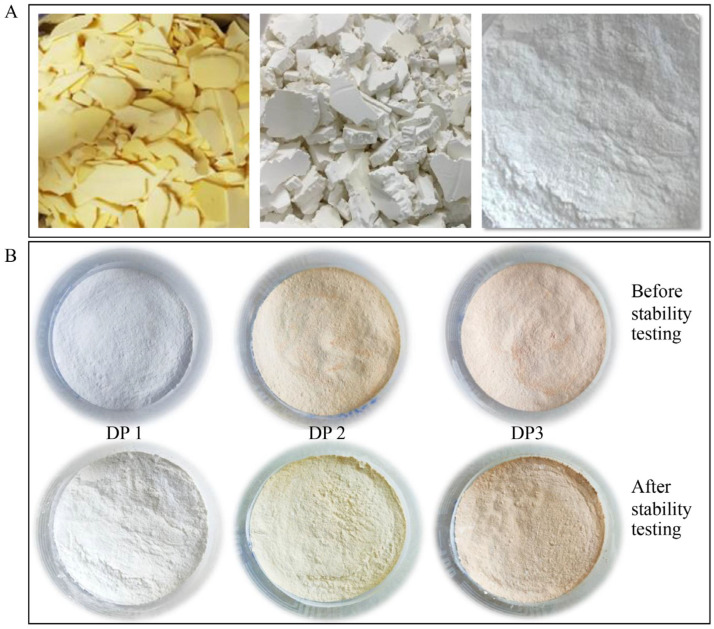
Starch powder extraction from fresh *D. hispida*. The fresh *D. hispida* tubers were cut into small pieces and subjected to the extraction method to obtain the powdered starch (**A**). Three dusting powder (DP) formulations before and after accelerated stability testing (DP 1, dusting powder base; DP 2, dusting powder base with added *G. glabra* extract; and DP 3, dusting powder base and added *G. glabra* extract and a coloring agent) (**B**).

Following the extraction of 8.8 kg of fresh *D. hispida*, 1.1 kg of powder (12.5% *w*/*w*) was obtained. The settle starch layer did not contaminate the alkaloid (dioscorine) or the steroid saponin (diosgenin). The starch powder had a white color with a smooth and fine texture (Figure 1A).

The main objective of this study was the evaluation of natural powder for use as an ingredient in cosmetics, such as DP. In this investigation, three distinct formulas were used to create DP from *D. hispida* starch. The dusting powder base formula was named DP 1. Dusting powder formulation No. 2 (DP 2) included *G. glabra* extract. Dusting powder formulation No. 3 (DP 3) included *G. glabra* extract along with Uthaithip liquid, a safe coloring ingredient that is often used in Thailand (Figure 1B).

### 3.4. SEM Study of Starch Particle Morphology

**Figure 2:**
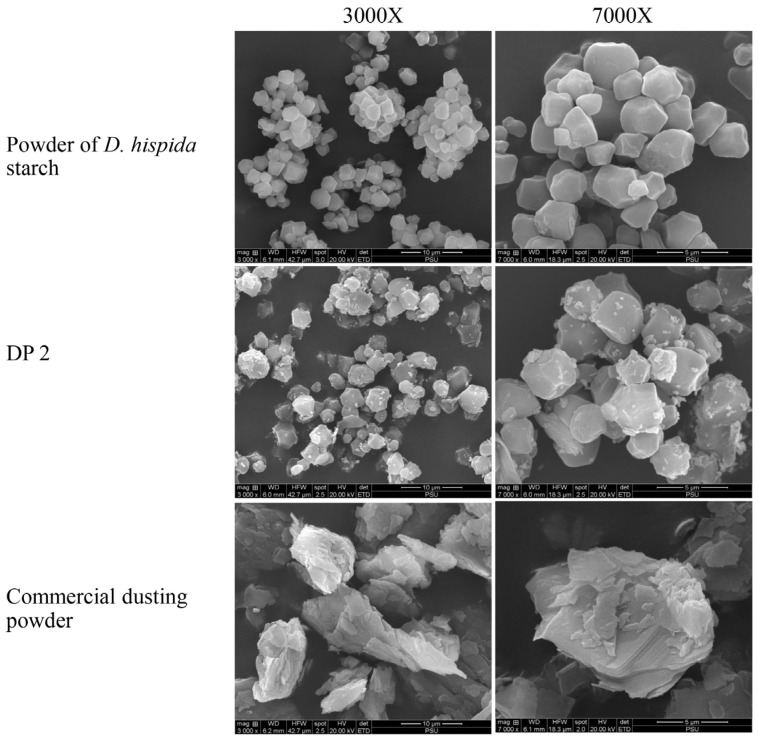
Scanning electron microscopy (SEM) images taken from the surface of powder of *D. hispida* starch, dusting powder formulation No. 2 (DP 2), and commercial dusting powder at 3000× and 7000×.

Scanning electron microscopy (SEM) analysis of the granules of samples revealed that the *D. hispida* starch was polygonal, had a smooth surface, and exhibited a particle size of approximately 2 μm to 4 μm. The particles of DP 2 were homogeneous and had attachments from the other ingredients. Furthermore, compared to the *D. hispida* starch, the DP 2 particles were more equally distributed. SEM images of the commercial DP revealed that the talc particles were about 5 μm to 15 μm in size and appeared as flat, tabular crystals (Figure 2).

### 3.5. Isolation of Dragon Fruit Pectin and Sleeping Mask (SM) Formulation

**Figure 3:**
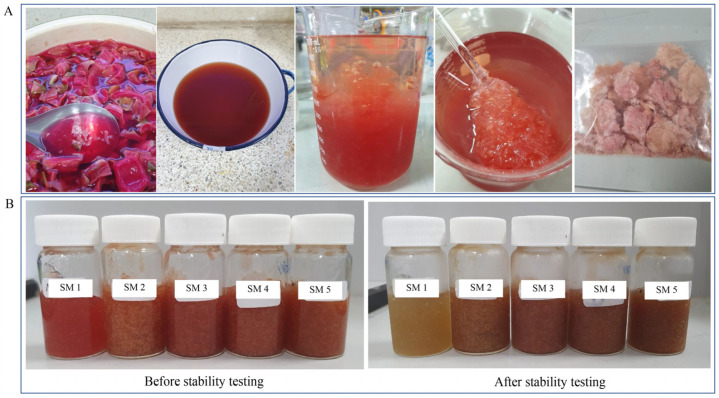
Pectin extracts from the dragon fruit peels. The peels were boiled in water. Absolute ethanol was added after the liquid portion had been separated and allowed to cool at 4 °C for an hour. Pectin was precipitated, allowed to dry at room temperature, and then dried using the freeze-drying technique (**A**). Five sleeping mask (SM) formulations before and after accelerated stability testing (SM 1, sleeping mask base; SM 2, sleeping mask base + *G. glabra* extract (1%); SM 3, sleeping mask base + *G. glabra* extract (1%) + *D. hispida* extract (0.5%); SM 4, sleeping mask base + *G. glabra* extract (1%) + *D. hispida* extract (1%); SM 5, sleeping mask base + *G. glabra* extract (1%) + *D. hispida* extract (2%) (**B**).

In this investigation, 1 kg of fruit peels yielded 14.15 g (1.42% *w*/*w*) of pectin. After freeze-drying, the pectin’s properties were red, and it turned into pectin powder when ground. When pectin was tested for swelling in water, it showed a red color and a consistently swelled, gel-like texture without the stickiness, and was odorous like dragon fruit peel. Its viscosity was 18,720 cps and its pH was 5.59 (Figure 3A).

The sleeping mask formulation was made using pectin from dragon fruit peel. It was discovered that 0.75 g of pectin was the appropriate amount to use for the basic formulation (SM 1) and that *G. glabra* extract (1%), which possesses tyrosinase inhibitory action, was added in SM 2. In the formulation development process, the inclusion of *D. hispida* extract—an extract with elastase inhibitory activity—at varying concentrations of 0.5% (SM 3), 1% (SM 4), and 2% (SM 5) took place in the following stage (Figure 3B).

### 3.6. Stability Study

#### 3.6.1. Characteristics of Dusting Powder (DP) Formulation

For the three DP formulations, the freeze–thaw stability was tested within six cycles to withstand undesired physical changes in starch. As shown in Table 5 and Figure 1B, the color, odor, and average particle size did not significantly change after the freeze–thaw cycles. This indicates that all three of the DP formulations have the ability to withstand accelerated conditions.

The static angle of repose values for the DP formulations are shown in Table 6. When the results were compared to Carr’s classification (Table 2), it was clear that DP 2 had fair flowability. Passable flowability was exhibited by DP 1. Prior to stability testing, DP 3 had only fair flowability; however, following that, it showed good flowability.

This study investigated whether *G. glabra* in DPs decreased tyrosinase activity using a mushroom tyrosinase activity assay. Table 6 shows that the two positive controls—kojic acid and the *A. lakoocha* water extract—had good stability in their inhibitory activity against tyrosinase. After the accelerated stability test, the enzyme inhibitory activity of kojic acid decreased from 81.27 ± 1.00% to 74.23 ± 5.26%, whereas the water extract from *A. lakoocha* showed no variation. After accelerated stability testing, the *G. glabra* extract showed tyrosinase inhibitory activity of 55.74 ± 1.10%. Compared to the *G. glabra* extract alone, this extract in DPs showed greater stability. When accelerated stability testing was performed, they exhibited tyrosinase inhibitory activity of 69.35 ± 2.58% and 67.90 ± 2.23% for DP 2 and DP 3, respectively.

#### 3.6.2. Characteristics of Sleeping Mask (SM) Formulation

This study developed pectin from dragon fruit peel to replace carbopol 934. The characteristics of the SM formulation before and after the stability test are shown in Table 7. SM 1 was the mask base, which had a gel-like appearance and a red color from the dragon fruit peel. However, the color did not stabilize because, after the acceleration process, the mask turned yellowish-white. The SM 2–SM 5 displayed a deep red color; however, after the acceleration procedure, the color changed slightly to reddish-brown. All SM formulations had a gel-like texture, spread easily, and were non-sticky. They had the odor of a coloring agent. The viscosity of SM 2–SM 5 increased after the acceleration process except for SM 1, while the pH value did not change.

Two enzyme types—tyrosinase and elastase—were the focus of the SM formulations. The presence of *G. glabra* extract in formulas SM 2–SM 5 had an effect on the tyrosinase enzyme. All formulas were proven to be effective at inhibiting the tyrosinase enzyme after preparation. During the acceleration procedure, the effect of the *G. glabra* extract was only slightly diminished, but the percentages of inhibition were more than 80.

The elastase enzyme plays a role in skin aging. Because *D. hispida* extract inhibited the elastase enzyme, it was chosen for this investigation to be included in the sleeping mask recipe. All formulas (SM 3–SM 5) containing *D. hispida* extract showed good elastase inhibitory effects during the experiment, even after the acceleration process.

### 3.7. Sensitization Dermal Toxicity 

**Figure 4:**
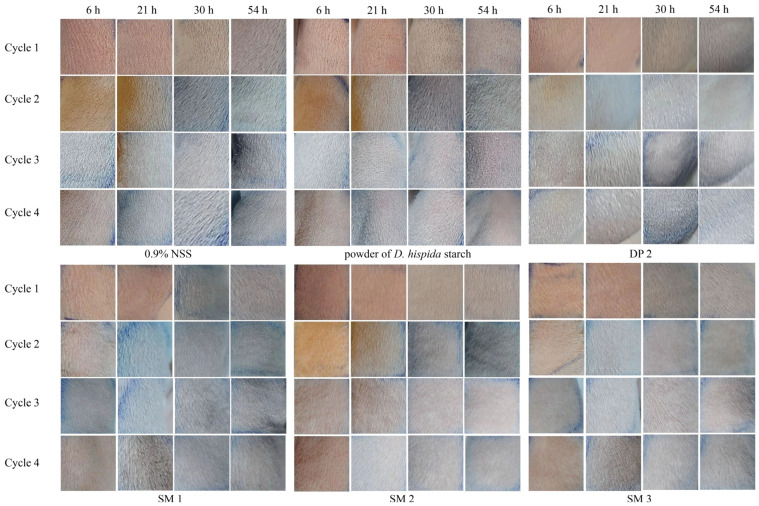
Representative images of guinea pig skin group with negative control (0.9% NSS), powder of *D. hispida* starch, DP 2, SM 1, SM 2, and SM 3 of 4 cycles at 6, 21, 30, and 54 h.

This study assessed the possibility that DP and SM formulations could irritate or trigger allergic reactions when they come into contact with guinea pig skin. Table 8 displays the average sensitization dermal toxicity level values from four cycles, challenging for each sample and control. It has been noted that 0.9%NSS and DP 2 produced level 1, which indicated no skin irritation or erythema in guinea pigs, comparable to the control group. The powder of *D. hispida* starch demonstrated well-defined erythema to moderate erythema categories (levels 2 to 3), indicating redness over more than half of the test area. The skin irritation of level 2 guinea pigs resulted in redness in all three SM formulas, including SM 1 (sleeping mask base), SM 2 (formula with 1% *G. glabra* extract), and SM 3 (the formula with 1% *G. glabra* extract and 0.5% *D. hispida* extract) (Figure 4).

## 4. Discussion

Herbal cosmetics are formulations made with phytochemicals derived from one or more plants. The desire for natural materials in cosmetic preparations has been driven by the common belief that cosmetics based on chemicals are unhealthy for people. Herbal cosmetics can be grouped into the following categories: cosmetics for enhancing the appearance of facial skin, hair growth and care, shampoos, soaps, powders, and perfumery [28]. This investigation was motivated by the abundance of *D. hispida* in southern Thailand. The authors conducted a study and found that it can be used as a cosmetic, like DP, or an ingredient in SM, rather than just as a food ingredient.

*D. hispida* or wild yam is widely found throughout Southeast Asia [29]. Dioscorine, a highly harmful water-soluble alkaloid, is present in its tuber. This tuber is poisonous by nature, but with the right processing, it can become edible. Boiling, roasting, or soaking the tuber in running water for 7 to 14 days is the traditional method of detoxification of tubers [30]. Therefore, before preparing the starch powder for cosmetic application, it is crucial to eliminate toxins from *Dioscorea*. The authors used processes like the locals do, such as washing the *Dioscorea* tuber under running water, steeping it in the water, and centrifugation of the settled starch layer. Then, we verified that the toxins were removed from the starch layer by using the Liebermann–Burchard test and the general test of alkaloids. This study found that the *D. hispida* starch content was about 12.5% *w*/*w,* which was lower than that reported by Hazrati et al. [12]. However, there was no alkaloid or steroid saponin contamination. After oven drying, this starch turned into a tiny, white powder that was ready for further development. Starch granules can be broadly classified into four types based on their botanical origins: spherical, elliptic, polygonal, and irregular [31]. The shapes and sizes of the starch granules from the various *Dioscorea* species varied. The granule forms of *D. rotundata*, *D. alata*, and *D. cayenensis* were comparable and primarily oval, ellipsoidal, and round. The granules of *D. bulbifera* starch had a triangular shape, whereas those of *D. dumetorum* had a hexagonal or polyhedral shape. In this study, the white *D. hispida* starch granules revealed a polyhedral shape, which ranged in size from 2 µm to 4 µm. These dimensions were similar to those previously reported [12,14]. Lindeboom et al. [32] classified starch granules into four groups: large (>25 µm), medium (10–25 µm), small (5–10 µm), and very small (<5 µm). The study found that the granules of *D. hispida* were very small in size. The morphological characteristics show that the starch granules that were extracted from this process are normal in shape and show no signs of damage. Thus, this investigation has shown that water extraction, centrifugation, and oven drying are suitable methods for the isolation of plant starch.

Talc is a common magnesium silicate mineral that is used in paper, ceramics, mouthwash, and even personal care products like baby powder. Additionally, it is the cause of an uncommon and difficult-to-identify kind of pneumoconiosis [33]. Talc can actually cause lung damage. Particularly, workers with frequent industrial exposure to talc are often found to have talc-related illnesses such as talc-silicosis and talc-asbestosis [34]. Although it is extremely uncommon, some patients still have lung damage as a result of inhaling talc from cosmetic powder [33]. There is currently demand in the cosmetics sector for the use of safe, natural starch as a talcum replacement. Therefore, instead of using talcum powder, the researchers created DP with starch from *D. hispida* and added *G. glabra* extract to boost its efficacy. According to the experiments conducted on the formulations of DP, all three formulations showed good stability when evaluated for stability in accelerated conditions, suggesting that the size, color, and odor of the formulations did not change. They also demonstrated good flowability. When examining the ability to suppress the tyrosinase enzyme activity of the *G. glabra* extract, it was discovered that their activity was less stable than that of the extract combined with DP 2. This could be because the ingredients in the DP recipe contributed to the extract and stability in DP.

Degeneration of the skin layer (dermis and epidermis) is what causes skin aging. Environmental factors, hormone imbalances, and sun exposure are some of the triggers that might contribute to accelerated aging. Signs of skin aging include dry skin, wrinkles, sagging, and the development of benign tumors [35]. Skin aging is caused by changes in the elastic network and collagen loss at the tissue level. The enzyme matrix metalloproteinases and elastase are involved in these tissue-level alterations [36]. Typical conditions require elastase and metalloproteinase activity to break down foreign proteins in the extracellular matrix (ECM) during neutrophil phagocytosis in order to facilitate tissue repair. However, in terms of anti-aging, finding elastase inhibitors may be helpful in preventing sagging and the loss of skin elasticity [37]. Apart from starch, which is a crucial component, the genus *Dioscorea* has also been found to contain active compounds that can be used in the cosmetics industry. The extract of *D. villosa* has anti-collagenase activity, which enhances the possibility of its application in anti-aging products and generally to combat skin degenerative diseases [38]. The ethanol extract of *D. hispida* in this investigation demonstrated anti-elastase activity. Because of this, the extract from *D. hispida* was utilized as an ingredient in SM formulation, which was made from dragon fruit peel pectin instead of synthetic materials. There were five formulas used to prepare SM: the sleeping mask base (SM 1), the formula with 1% *G. glabra* extract (SM 2), and three formulas that were supplemented with 1% *G. glabra* extract and *D. hispida* extract in varying amounts: 0.5% (SM 3), 1% (SM 4), and 2% (SM 5). Sleeping masks are gel-like, non-separating, and non-sticky masks whereby no clumping occurs and are absorbable via the skin. The pH of all formulas was approximately 5. Nevertheless, the red color of dragon fruit peel pectin did not stabilize. As can be observed, the red color of pectin was eliminated when SM 1 was accelerated. Dragon fruit peel pectin contains a red pigment called betacyanin, which is a harmless, naturally occurring color that has been utilized in food and cosmetics. Betacyanin was stable between pH 4 and 6, although it degraded easily in the presence of light, oxygen, and high temperatures [39]. For this reason, when making sleeping masks, more coloring agent was included in this study. After stability testing, the color of the *G. glabra* and *D. hispida* extracts may have been related to the deeper color of the SM 2–SM 5 formulas. The texture, odor, and pH were identical. However, the viscosity simply increased because there was no stirring [40].

Tyrosinase is an oxidase enzyme that contains copper and is involved in the first stages of melanin synthesis in many organisms. The rate-limiting step of melanin production, the oxidation of the substrate, L-tyrosine or L-3,4-dihydroxyphenylalanine (L-DOPA) to DOPA-quinone, is catalyzed by this enzyme. Numerous dermatological conditions, including wrinkles, melasma, freckles, lentigo, ephelides, nevus, melanoma, and skin aging spots, are caused by an excess of melanin pigments [41,42]. Consequently, the *G. glabra* extract was chosen for this investigation as an ingredient in SM and DP formulations due to its potent tyrosinase enzyme inhibitory properties [43,44]. *G. glabra* compounds that have been shown to inhibit tyrosinase activity include licochalcone A, glabridin, glabrene, isoliquiritigenin, and liquiritin [45,46]. Glabridin, an isoflavonoid, is isolated from *G. glabra* roots. In previous reports, glabridin inhibited the mushroom tyrosinase enzyme with an IC_50_ value of 0.08 µg/mL. It also inhibited murine melanoma (B16F1) intracellular tyrosinase, showing an IC_50_ value of 0.69 µg/mL. Moreover, glabridin did not affect melanin synthesis in a zebrafish model [43]. Other compounds, including isoliquiritigenin and glabrene, reduced the activities of both monophenolase and diphenolase tyrosinase. They exhibited IC_50_ values of 3.5 and 8.1 µM when using tyrosine as the substrate [47].

*Dioscorea* is a plant that has applications in an abundance of fields, including food, cosmetics, and pharmaceuticals. According to earlier investigations, the ethanol extract of *D. hispida* could inhibit the elastase enzyme at a concentration of 2 mg/mL (80.71 ± 1.38%), with an IC_50_ value of 15.13 µg/mL. Therefore, this study focuses on the cosmetic field because *D. hispida* extract was chosen as an ingredient in the SM formulations. Prior research has demonstrated that *Dioscorea* extract not only promoted anti-aging but also promoted cell proliferation and skin hydration [48]. Diosgenin derived from *D. villosa* or *D. composita* extracts was used as a supplementary dietary supplement in 2009. Using a human 3D skin comparable model in vitro, diosgenin’s effectiveness against skin aging was demonstrated. Tada et al. [49] reported that it boosted intracellular cAMP levels, bromodeoxyuridine absorption, and DNA synthesis in adult human keratinocytes. According to these findings, diosgenin may have an impact on keratinocyte proliferation repair in aging skin. Likewise, *T. terrestris* extract high in saponins was found to exhibit inhibitory activity on collagenase and elastase in an in vitro assay by Lee et al. [50]. According to GC-MS analysis, the main components of the *D. hispida* extracts were fatty acids, such as 9,12-octadecadienoic acid (Z,Z)- (linoleic acid). Natural fatty acids are crucial for preserving the epidermal barrier and reducing inflammation. Alpha-linolenic acid (ALA) and linoleic acid (LA), the two primary necessary fatty acids, have the capacity to repair the skin barrier. These fatty acids are mostly found in flaxseed, walnut, grape seed, safflower, sunflower, blackcurrant, evening primrose, and borage oil [51].

Some *Dioscorea* species have been demonstrated to contain the toxic alkaloid dioscorine as a toxic principle. Dioscorine causes the nervous system to become fatally paralyzed when a 100 g piece of tuber is consumed [52]. Similarly, it has been found that the main allergen in many Dioscoreaceae plants is histamine, which causes moderate inflammation and itching [53]. Therefore, the application of *Dioscorea* starch and extract as an ingredient in DP and SM requires allergy testing, especially skin allergy testing. In this research, guinea pig skin developed skin allergies at levels 2 to 3 after the powder of *D. hispida* starch was applied, which can result in redness of their skin. However, the guinea pigs did not exhibit any adverse symptoms when DP 2 was applied. The results indicate that *D. hispida* starch has a potential component that may be employed as a cosmetic application since its properties show easy extraction, good stability, safety, and low costs. The SM formulations, including SM 1 (sleeping mask base), SM 2 (formula + 1% *G. glabra*), and SM 3 (base formula + 1% *G. glabra* extract + 0.5% *D. hispida* extract), showed skin allergies at level 2 (not severe), which can result in redness of the skin. Even without adding the plant extract to the SM formula base, an allergic reaction was found in the study. The acidity of the product may be the cause of this. The skin pH criteria range is between 4.5 and 6.5. While a very acidic pH may irritate the skin, an extremely alkaline pH can cause the skin to become dry and scaly [54].

## 5. Conclusions

*D. hispida* starch from the south of Thailand cultivars was successfully separated with water. The starch showed a white color in a fine powder. The morphology was polyhedral in shape, had a smooth surface, and exhibited small granules. *D. hispida* starch was used in DP formulations to provide a smooth texture. The color, odor, size, and flowability of dusting powder formulations had stability when subject to six cycles of freeze–thaw procedures. The DP 2 formulation, which was supplemented by 8% *G. glabra* extract, showed the highest tyrosinase inhibitory activity after the stability test. Additionally, this formula did not affect guinea pig skin. In addition, *D. hispida* ethanolic extract exhibited potential anti-elastase activity. This activity remained stable in SM formulations. Therefore, *D. hispida* could potentially be utilized as an alternative to talcum in the production of dusting powder bases and an ingredient supplementing sleeping mask gel.

## Figures and Tables

**Table 1:** Formulations of *D. hispida* dusting powder (DP) (100 g).

Ingredients	Function	Formular		
		DP 1	DP 2	DP 3
*D. hispida* powder (g)	Powder base	86	78	78
*G. glabra* extract (g)	Whitening agent	-	8	8
Zinc oxide (g)	Adhesive agent	7.5	7.5	7.5
Magnesium stearate (g)	Absorbance agent	1	1	1
Kaolin (g)	Lubricant	5	5	5
Phenoxyethanol (g)	Preservative	0.5	0.5	0.5
Uthaithip^®^ Solution	Coloring agent	-	-	q.s.

q.s.; as much as suffices.

**Table 2:** Formulations of sleeping mask (SM) from pectin of dragon fruit peels (25 mL).

Ingredients	Function	Formular				
		SM 1	SM 2	SM 3	SM 4	SM 5
Glycerine (mL)	Humectant	2	2	2	2	2
Pectin of dragon fruit peels (g)	Gelling agent	0.75	0.75	0.75	0.75	0.75
Propylene glycol (mL)	Humectant	1	1	1	1	1
Sodium benzoate (g)	Preservative	0.125	0.125	0.125	0.125	0.125
*G. glabra* extract (g)	Whitening agent	-	0.25	0.25	0.25	0.25
*D. hispida* extract (g)	Anti-aging agent	-	-	0.125	0.25	0.50
Uthaithip^®^ Solution	Coloring agent	-	q.s.	q.s.	q.s.	q.s.
Distilled water q.s.to	Solvent	25	25	25	25	25

q.s.; as much as suffices.

**Table 3:** Flow properties and angle of repose.

Flow Ability	Angle of Repose (Degrees)
Excellent	25–30
Good	31–35
Fair (aid not needed)	36–40
Passable (may hang up)	41–45
Poor (must agitate, vibrate)	46–55
Very poor	56–65
Very, very poor	>66

**Table 4:** Criteria for erythema formation and edema formation scoring values in sensitization dermal toxicity test.

Skin Responses	Score
*Erythema formation*	
No erythema	0
Very slight erythema (barely perceptible)	1
Well-defined erythema	2
Moderate erythema (Redness occurs over more than half of the test area)	3
Severe erythema (Redness appeared over the entire test area; redness from scabs)	4
*Edema formation*	
No edema	0
Very slight edema (barely perceptible)	1
Slight edema (edges of area well defined by definite raising)	2
Moderate edema (raised approximately 1·0 mm)	3
Severe edema (raised more than 1·0 mm and extending beyond exposure area)	4

**Table 5:** Phytochemical compounds of *Dioscorea hispida* ethanolic extract from GC-MS analysis.

No.	RT	Name of Compound	Molecular Formula	Match Factor	Peak Area%
1	7.60	4-Hydroxy-2,5-dimethyl-3(2H)-furanone	C_6_H_8_O_3_	90.70	0.39
2	8.93	4H-Pyran-4-one, 2,3-dihydro-3,5-dihydroxy-6-methyl-	C_6_H_8_O_4_	97.40	2.77
3	11.80	1-Methyl-3,4-dihydroisoquinoline	C_10_H_11_N	94.90	1.43
4	14.08	Phenol, 2,4-bis(1,1-dimethylethyl)-	C_14_H_22_O	93.50	0.28
5	21.06	Hexadecanoic acid, methyl ester	C_17_H_34_O_2_	91.90	0.13
6	22.22	n-Hexadecanoic acid	C_16_H_32_O_2_	90.80	0.29
7	22.75	Hexadecanoic acid, ethyl ester	C_18_H_36_O_2_	98.50	2.84
8	24.86	9,12-Octadecadienoic acid (Z,Z)-, methyl ester	C_19_H_34_O_2_	94.10	0.22
9	26.08	9,12-Octadecadienoic acid (Z,Z)-	C_18_H_32_O_2_	94.70	6.51
10	26.36	Linoleic acid ethyl ester	C_20_H_36_O_2_	96.70	5.72
11	26.52	(E)-9-Octadecenoic acid ethyl ester	C_20_H_38_O_2_	91.50	1.26
12	27.10	Octadecanoic acid, ethyl ester	C_20_H_40_O_2_	92.10	0.95
13	35.13	(Z,Z)-9,12-octadeca-dienoic acid, 2,3-dihydroxy-propyl	C_21_H_38_O_4_	91.90	3.89
14	41.27	Cholest-5-en-3-ol (3.beta.)-	C_27_H_46_O	94.00	0.24
15	41.41	dl-.alpha.-Tocopherol	C_29_H_50_O_2_	97.00	1.17
16	42.68	Campesterol	C_28_H_48_O	95.90	3.40
17	43.11	Stigmasta-5,22-dien-3-ol, (3.beta.,22E)-	C_29_H_48_O	93.40	6.41
18	43.77	.gamma.-Sitosterol	C_29_H_50_O	97.30	0.73

**Table 6:** Evaluation parameters of dusting powder (DP).

Sample	Characteristics	
	Before Stability Testing	After Stability Testing
	Color and odor	
DP 1	White powder color with smooth and fine appearance with *Dioscorea* odor	White powder color with smooth and fine appearance with *Dioscorea* odor.
DP 2	Light yellow powder color with smooth and fine, with *G. glabra* extract odor	Light yellow powder color. Slightly clumped with *G. glabra* extract odor
DP 3	Pinkish-orange powder color with smooth and fine appearance with a mild perfume of coloring agent odor	Pinkish-orange powder color with smooth and fine appearance with a mild perfume of coloring agent odor.
	Particle size	
DP 1	2.31 ± 0.48 µm	2.35 ± 0.43 µm
DP 2	2.23 ± 0.34 µm	2.29 ± 0.49 µm
DP 3	2.26 ± 0.41 µm	2.32 ± 0.42 µm
	Angle (°)/Flow property	
DP 1	45/passable	41.0/passable
DP 2	40/fair	36.5/fair
DP 3	39.3/fair	35.0/good
	Tyrosinase inhibition (%) (20 µg/mL)	
DP 1	-	-
DP 2	71.53 ± 2.23	69.35 ± 2.58
DP 3	70.60 ± 1.89	67.90 ± 2.23
*G. glabra* extract	69.84 ± 1.94	55.74 ± 1.10
kojic acid	81.27 ± 1.00	74.23 ± 5.26

**Table 7:** Evaluation parameters of sleeping mask.

Sample	Characteristics	
	Before Stability Testing	After Stability Testing
	Color and odor	
SM 1	Red with a fine appearance of dragon fruit peel odor	Yellow white with a fine appearance of dragon fruit peel odor
SM 2	Light-red with a mild perfume of coloring agent odor	Red-brown with a mild perfume of coloring agent odor
SM 3	Dark-red with a mild perfume of coloring agent odor	Red-brown with a mild perfume of coloring agent odor
SM 4	Dark-red with a mild perfume of coloring agent odor	Red-brown with a mild perfume of coloring agent odor
SM 5	Dark-red with a mild perfume of coloring agent odor	Red-brown with a mild perfume of coloring agent odor
	Texture	
SM 1	Gel-like texture, easy to apply on skin, and no separation	Gel-like texture, easy to apply on skin, and no separation
SM 2	Gel-like texture, easy to apply on skin, and no separation	Gel-like texture, easy to apply on skin, and no separation
SM 3	Gel-like texture, easy to apply on skin, and no separation	Gel-like texture, easy to apply on skin, and no separation
SM 4	Gel-like texture, easy to apply on skin, and no separation	Gel-like texture, easy to apply on skin, and no separation
SM 5	Gel-like texture, easy to apply on skin, and no separation	Gel-like texture, easy to apply on skin, and no separation
	Viscosity (cPs)	
SM 1	11,233	11,147
SM 2	11,290	12,407
SM 3	11,891	12,050
SM 4	11,899	13,107
SM 5	11,903	12,870
	pH	
SM 1	5.39	5.39
SM 2	5.36	5.34
SM 3	5.34	5.35
SM 4	5.37	5.37
SM 5	5.36	5.36
	Tyrosinase inhibition (%) (20 µg/mL)	
SM 1	-	-
SM 2	82.85 ± 0.99	80.24 ± 1.75
SM 3	100.29 ± 1.60	84.24 ± 1.68
SM 4	94.12 ± 1.38	88.97 ± 1.83
SM 5	97.79 ± 1.73	86.50 ± 0.91
*G. glabra* extract	74.49 ± 0.14	88.12 ± 0.77
kojic acid	74.63 ± 0.44	86.18 ± 0.97
	Elastase inhibition (%) (2 mg/mL)	
SM 1	-	-
SM 2	-	-
SM 3	89.82 ± 2.10	83.26 ± 3.64
SM 4	83.92 ± 0.95	81.96 ± 2.09
SM 5	89.94 ± 1.04	80.72 ± 2.21
*D. hispida* extract	81.76 ± 1.51	75.02 ± 3.35
EGCG	98.99 ± 0.72	98.96 ± 0.17

**Table 8:** Classification of sensitization dermal toxicity test result, N = 6.

Formula	Cycle 1	Cycle 2	Cycle 3	Cycle 4	Level	Type of Skin Reaction
0.9% NSS	1.04	0	0	0	Level 1	No irritated reaction
*D. hispida* starch	14.58	29.16	23.95	19.79	Level 2–3	Not severe to moderate
DP 2	0	0	0	0	Level 1	No irritated reaction
SM 1	3.13	8.33	15.65	16.66	Level 2	Not severe
SM 2	8.33	10.41	18.75	21.87	Level 2	Not severe
SM 3	10.41	12.50	20.83	18.75	Level 2	Not severe

## Data Availability

Data are contained within the article and Supplementary Materials.

## Supplementary Materials

The following supporting information can be downloaded at: www.mdpi.com/article/10.3390/life15020151/s1. Figure S1: All GC-MS data of *D. hispida* extract.

## References

[1] Al-Khayri J.M., Rashmi R., Toppo V., Chole P.B., Banadka A., Sudheer W.N., Nagella P., Shehata W.F., Al-Mssallem M.Q., Alessa F.M. (2023). Plant Secondary Metabolites: The Weapons for Biotic Stress Management. Metabolites.

[2] Korkina L., Kostyuk V., Potapovich A., Mayer W., Talib N., De Luca C. (2018). Secondary Plant Metabolites for Sun Protective Cosmetics: From Pre-Selection to Product Formulation. Cosmetics.

[3] Chen D., Mubeen B., Hasnain A., Rizwan M., Adrees M., Naqvi S.A.H., Iqbal S., Kamran M., El-Sabrout A.M., Elansary H.O. (2022). Role of Promising Secondary Metabolites to Confer Resistance Against Environmental Stresses in Crop Plants: Current Scenario and Future Perspectives. Front. Plant Sci..

[4] Kabera J.N., Semana E., Mussa A.R., He X. (2014). Plant secondary metabolites: Biosynthesis, classification, function and pharmacological properties. J. Pharm. Pharmacol..

[5] Gyawali R., Paudel P.N. (2022). Herbal remedies in Cosmeceuticals formulation: A review on Nepalese perspectives. Annapurna J. Health Sci..

[6] Luo G.F., Podolyan A., Kidanemariam D.B., Pilotti C., Houliston G. (2022). A review of viruses infecting Yam (*Dioscorea* spp.). Viruses.

[7] Ono Y., Thiphaphorn N., Thamthurasan W., Unartngam J., Okane I. (2021). Two *Dioscorea* rust fungi found in Thailand. Jpn. J. Mycol..

[8] Liu Y., Li H., Fan Y., Man S., Liu Z., Gao W., Wang T. (2016). Antioxidant and antitumor activities of the extracts from Chinese Yam (*Dioscorea opposite* Thunb.) flesh and peel and the effective compounds. J. Food Sci..

[9] Zeng X., Liu D., Huang L. (2021). Metabolome profiling of eight Chinese yam (*Dioscorea polystachya* Turcz.) varieties reveals metabolite diversity and variety specific uses. Life.

[10] Obidiegwu J.E., Lyons J.B., Chilaka C.A. (2020). The *Dioscorea* Genus (Yam)—An appraisal of nutritional and therapeutic potentials. Foods.

[11] Epping J., Laibach N. (2020). An underutilized orphan tuber crop-Chinese yam: A review. Planta.

[12] Hazrati K.Z., Sapuan S.M., Zuhri M.Y.M., Jumaidin R. (2021). Extraction and Characterization of Potential Biodegradable Materials Based on *Dioscorea hispida* Tubers. Polymers.

[13] Oni S.O. (2020). Physical characteristics and nutritional analysis of native and chemically modified starches obtained from Yam (*Dioscorea rotundata*) and Cassava (*Manihot esculeta*) Tubers. J. Food Nutr..

[14] Ashri A., Yusof M.S.M., Jamil M.S., Abdullah A., Yusoff S.F.M., Arip M.N.M., Lazim A.M. (2014). Physicochemical characterization of starch extracted from Malaysian wild yam (*Dioscorea hispida* Dennst.). Emir. J. Food Agric..

[15] Sheikh F.A., Baheti M.G. (2020). Formulation and evaluation of antimicrobial dusting powder. Int. J. Pharm. Pharm. Sci..

[16] Chang H., Zhang J., Xia J., Kang C., Yan Y. (2022). Influence of waxy proteins on wheat resistant starch formation, molecular structure and physicochemical properties. Food Chem..

[17] Jiang Y., Liu L., Wang B., Yang X., Chen Z., Zhong Y., Zhang L., Mao Z., Xu H., Sui X. (2019). Polysaccharide-based edible emulsion gel stabilized by regenerated cellulose. Food Hydrocoll..

[18] Rincón A.M., Pérez R.M.N., Reyes A., Romero A., Orfila L., Padilla F.C. (2005). ‘Guapo’ (*Myrosma cannifolia*) starch: A natural product with potential use in cosmetic formulations. Int. J. Cosmet. Sci..

[19] Boonme P., Aporn M., Khwankaew S., Pichayakorn W., Prapruti P., Boromthanarat S. (2009). Feasibility study of sago starch for perfumed and cooling body powders. Cosm. Toil.

[20] Nilforoushzadeh M.A., Amirkhani M.A., Zarrintaj P., Salehi Moghaddam A., Mehrabi T., Alavi S., Mollapour Sisakht M. (2018). Skin care and rejuvenation by cosmeceutical facial mask. J. Cosmet. Dermatol..

[21] Morganti P., Morganti G., Chen H.D., Gagliardini A. (2019). Beauty mask: Market and environment. J. Clin. Cosmet. Dermatol..

[22] Masdar N.D., Roslan R.A.B., Hasan S.B., Kamal M.L. (2020). Determination of antioxidant from UBI gadong tubers for facial soap bar. Charting the Sustainable Future of ASEAN in Science and Technology: Proceedings of the 3rd International Conference on the Future of ASEAN (ICoFA) 2019-Volume 2.

[23] Lim T.K. (2016). *Dioscorea* *hispida*. Edible Medicinal and Non-Medicinal Plants.

[24] Neag E., Stupar Z., Torok A.I., Surupaceanu I., Senila M., Cadar O. (2022). Exploring the properties of micronized natural zeolitic volcanic tuff as cosmetic ingredient. Materials.

[25] Carr R.L. (1965). Evaluating flow properties of solids. Chem. Eng..

[26] Dej-adisai S., Parndaeng K., Wattanapiromsakul C. (2016). Determination of phytochemical compounds, and tyrosinase inhibitory and antimicrobial activities of bioactive compounds from *Streblus ilicifolius* (S Vidal) Corner. Trop. J. Pharm. Res..

[27] Lee K.K., Cho J.J., Park E.J., Choi J.D. (2001). Anti-elastase and anti-hyaluronidase of phenolic substance from Areca catechu as a new anti-ageing agent. Int. J. Cosmet. Sci..

[28] Sumit K., Vivek S., Sujata S., Ashish B. (2012). Herbal cosmetics: Used for skin and hair. Inven. J..

[29] Denham T., Iriarte J., Vrydaghs L. (2007). Rethinking Agriculture: Archaeological and Ethnoarchaeological Perspective.

[30] Hudzari R.M., Ssomad M.A.H.A., Rizuwan Y.M., Asimi M.N.N., Abdullah A.B.C., Fauzan M.Z.M. (2011). Modification of automatic alkaloid removal system for dioscorine. Int. J. Agron. Plant Prod..

[31] Xia Y., Gao W., Wang H., Jiang Q., Li X., Huang L., Xiao P. (2013). Characterization of tradition Chinese medicine (TCM) starch for potential cosmetics industry application. Starch-Stärke.

[32] Lindeboom N., Chang P.R., Tyler R.T. (2004). Analytical, biochemical and physicochemical aspects of starch granulesize, with emphasis on small granule starches: A review. Starch-Stärke.

[33] Cho A., Amirahmadi R., Ajmeri A., Deepak J. (2021). Pulmonary talcosis in the setting of cosmetic talcum powder use. Respir. Med. Case Rep..

[34] Jasuja S., Kuhn B.T., Schivo M., Adams J.Y. (2017). Cosmetic talc–related pulmonary granulomatosis. J. Investig. Med. High Impact Case Rep..

[35] Mukherjee P.K., Maity N., Nema N.K., Sarkar B.K. (2011). Bioactive compounds from natural resources against skin aging. Phytomedicine.

[36] Sittek L.M., Schmidts T.M., Schlupp P. (2023). Potential Application of a Wine Extract in Skin Care: How to Benefit from the Antibacterial, Antioxidant and Elastase Inhibiting Properties. J. Cosmet. Dermatol. Sci. Appl..

[37] Thring T.S., Hili P., Naughton D.P. (2009). Anti-collagenase, anti-elastase and anti-oxidant activities of extracts from 21 plants. BMC Complement. Altern. Med..

[38] Binic I., Lazarevic V., Ljubenovic M., Mojsa J., Sokolovic D. (2013). Skin ageing: Natural weapons and strategies. Evid.-Based Complement. Altern. Med..

[39] Sen R., Baruah A.M. (2023). Phenolic profile and pigment stability of *Hylocereus* species grown in North-East India. J. Food Compos. Anal..

[40] Tanjung Y.P. (2021). Formulation and evaluation of peel off gel facial mask from arabica coffee fruit peel extract (*Coffea arabica* L.). Arabica.

[41] Lee S.W., Kim J.H., Song H., Seok J.K., Hong S.S., Boo Y.C. (2019). Luteolin 7-Sulfate Attenuates Melanin Synthesis through Inhibition of CREB- and MITF-Mediated Tyrosinase Expression. Antioxidants.

[42] Hałdys K., Goldeman W., Jewgiński M., Wolińska E., Anger N., Rossowska J., Latajka R. (2018). Inhibitory properties of aromatic thiosemicarbazones on mushroom tyrosinase: Synthesis, kinetic studies, molecular docking and effectiveness in melanogenesis inhibition. Bioorg. Chem..

[43] Dej-adisai S., Koyphokaisawan N., Wattanapiromsakul C., Nuankaew W., Kang T.H., Pitakbut T. (2023). In Vitro, In Vivo, and In Silico Analyses of Molecular Anti-Pigmentation Mechanisms of Selected Thai Rejuvenating Remedy and Bioactive Metabolites. Molecules.

[44] Panzella L., Napolitano A. (2019). Natural and bioinspired phenolic compounds as tyrosinase inhibitors for the treatment of skin hyperpigmentation: Recent Advances. Cosmetics.

[45] Ebanks J.P., Wickett R.R., Boissy R.E. (2009). Mechanisms regulating skin pigmentation: The rise and fall of complexion coloration. Int. J. Mol. Sci..

[46] Nerya O., Vaya J., Musa R., Izrael S., Ben-Arie R., Tamir S. (2003). Glabrene and isoliquiritigenin as tyrosinase inhibitors from licorice roots. Agric. Food Chem..

[47] Zhu W., Gao J. (2008). The Use of Botanical Extracts as Topical Skin-Lightening Agents for the Improvement of Skin Pigmentation Disorders. J. Investig. Dermatol. Symp. Proc..

[48] Kim D.-S., Jeon B.-K., Mun Y.-J., Kim Y.-M., Lee Y.-E., Woo W.-H. (2011). Effect of *Dioscorea aimadoimo* on Anti-Aging and Skin Moisture Capacity. J. Physiol. Pathol. Korean Med..

[49] Tada Y., Kanda N., Haratake A., Tobiishi M., Uchiwa H., Watanabe S. (2009). Novel effects of diosgenin on skin aging. Steroids.

[50] Lee S., An H., Kim W., Lu X., Jeon H., Park H.W., Ha J., Cho J. (2021). Anti-aging Effect of Mixed Extract from Medicinal Herbs. Asian J. Beauty Cosmetol..

[51] Xie M., Jiang Z., Lin X., Wei X. (2024). Application of plant extracts cosmetics in the field of anti-aging. J. Dermatol. Sci. Cosmet. Technol..

[52] Bhandari M.R., Kawabata J. (2005). Bitterness and toxicity in wild yam (*Dioscorea* spp.) tubers of Nepal. Plant Foods Hum. Nutr..

[53] Kumar S., Das G., Shin H.S., Patra J.K. (2017). *Dioscorea* spp. (A Wild Edible Tuber): A study on its ethnopharmacological potential and traditional use by the local people of similipal biosphere reserve, India. Front. Pharmacol..

[54] Rahmawanty D., Yulianti N., Fitriana M. (2015). Formulation and evaluation of peel off face mask containing quercetin with gelatin and glycerin concentration variation. J. Media Farm..

