# Supplementary material for: Utility Assessment of Isolated Starch and Extract from Thai Yam (Dioscorea hispida Dennst.) for Cosmetic via In Vitro and In Vivo Studies
Source: Life (Basel). 2025 Jan 22;15(2):151. doi: 10.3390/life15020151 (PMC11856013; doi:10.3390/life15020151)
Supplement: Supplementary file 1 [file life-15-00151-s001.zip › life-3425824-supplementary.pdf]

Library Search Results - NonTarget Hits with Details

**Batch Path**	D:\MassHunter\GCMS\1\data\2567\0037_67\18-10-2023		
**Analysis File Name**	CompoundsIdentificationscore60up.uaf		
**Analyst Name**	admin	**Analysis Time**	10/19/2023 4:16:36 PM
**File Name**	4_0037_67DH_N401.D	**Path Name**	D:\MassHunter\GCMS\1\data\2567\0037_67\18-10-2023
**Sample Name**	DH	**Type**	Sample
**Acq. Method File**	0037_67split1_1M4	**Acq. Method Path**	D:\MassHunter\GCMS\1\methods\Method2567\
**Acq. Date-Time**	10/19/2023 10:10:52 AM	**Acq. Operator**	Waraporn
**Instrument Name**	GCMS	**Dil.**	1

Component RT	Compound Name	CAS#	Formula	Component Area	Match Factor	Estimated Conc.
3.4850	2,3-Butanediol	513-85-9	C4H10O2	1932696.0	61.3	
3.6967	Methylamine, N-(1-methylhexylidene)-	22058-71-5	C8H17N	1826203.1	62.0	
4.4409	2-Furanmethanol	98-00-0	C5H6O2	4384867.0	83.4	
4.7295	l-Alanine, N-methoxycarbonyl-, methyl ester	28819-00-3	C6H11NO4	10606193.1	65.3	
4.8129	2(3H)-Furanone, dihydro-	96-48-0	C4H6O2	7708453.3	61.9	
5.3647	1-Methoxy-2-methyl-3-butene	92808-02-1	C6H12O	48201852.7	63.1	
5.9036	5,7-Octadien-3-ol, 2,4,4,7-tetramethyl-, (E)-	77142-78-0	C12H22O	3406544.1	70.2	
6.3175	2,4-Dihydroxy-2,5-dimethyl-3(2H)-furan-3-one	10230-62-3	C6H8O4	28722700.4	89.3	
6.8659	Benzene, (chloromethyl)-	100-44-7	C7H7Cl	10578654.0	79.6	
7.2187	Benzeneacetaldehyde	122-78-1	C8H8O	4407857.0	64.9	
7.4818	Ethanone, 1-(1H-pyrrol-2-yl)-	1072-83-9	C6H7NO	743169.5	80.0	
7.6036	4-HYDROXY-2,5-DIMETHYL-3(2H)-FURANONE	3658-77-3	C6H8O3	10673020.6	90.7	
7.8923	2,4(1H,3H)-Pyrimidinedione, 5-methyl-	65-71-4	C5H6N2O2	7896437.9	77.0	
8.2773	N-Methylpyrrole-2-carboxylic acid	6973-60-0	C6H7NO2	1228634.7	66.9	
8.4248	2-acetyl-2-hydroxy-.gamma.-butyrolactone	135366-64-2	C6H8O4	16466342.4	76.3	
8.5724	Nonane	111-84-2	C9H20	3071066.7	61.0	
8.6750	1,3-Cyclohexadiene, 2-methyl-5-(1-methylethyl)-	99-83-2	C10H16	911529.2	71.9	
8.7777	2(1H)-Pyridinone, 6-methyl-	3279-76-3	C6H7NO	2428989.1	62.0	
8.9252	4H-Pyran-4-one, 2,3-dihydro-3,5-dihydroxy-6-methyl-	28564-83-2	C6H8O4	74766177.0	97.4	
9.4577	Benzoic acid	65-85-0	C7H6O2	1560779.8	83.2	
9.8234	[1,3]Diazepan-2,4-dione	75548-99-1	C5H8N2O2	1488346.5	68.0	
9.9496	5-Hydroxymethylfurfural	67-47-0	C6H6O3	26291663.3	75.4	
10.2275	(3E)-4-(1-CYCLOPENTEN-1-YL)-3-BUTEN-2-ONE	110845-85-7	C9H12O	4456417.5	76.0	
10.5227	1,3-Dioxan-5-ol, 2-pentadecyl-, acetate, trans-	30889-26-0	C21H40O4	450983.2	60.9	
10.7793	2-Propanamine, N-(phenylmethylene)-	6852-56-8	C10H13N	4652448.7	60.9	
10.9397	3-Piperidino-1,2-propanediol	4847-93-2	C8H17NO2	3298878.6	68.4	
11.2540	2-ALLYL-3,5-DIMETHYLPYRAZINE	55138-70-0	C9H12N2	5381094.2	67.9	
11.3759	2-Methoxy-4-vinylphenol	7786-61-0	C9H10O2	1838166.6	63.3	
11.6261	2-Naphthalenamine	91-59-8	C10H9N	2043539.7	88.9	
11.7416	9-Azabicyclo[3.3.1]nonan-3-one, 9-methyl-	552-70-5	C9H15NO	814653.5	78.8	
11.7993	1-Methyl-3,4-dihydroisoquinoline	2412-58-0	C10H11N	38658906.1	94.9	
11.9084	3-METHYL-5,6,7,8-TETRAHYDROISOQUINOLINE	37009-20-4	C10H13N	1758891.8	83.9	
12.0303	1-Methyl-3,4-dihydroisoquinoline	2412-58-0	C10H11N	6302685.8	92.7	
12.1778	2,7-Methanonaphthalen-3-amine, 1,2,3,4,4a,7,8,8a-octahydro-N-methyl-, (2.alpha.,3.alpha.,4a.beta.,7.alpha.,8a.beta.)-	1000186-49-9	C12H19N	12866996.5	78.9	
12.4344	3-Pyridinemethanol, 5-hydroxy-4,6-dimethyl-	61-67-6	C8H11NO2	15472788.5	82.8	
12.4793	N-(4-Methoxyphenyl)glycine	1000452-49-9	C9H11NO3	16111739.7	70.7	
12.7231	2-ethoxycarbonyl-5-oxo pyrrolidine	2000089-40-2	C7H11NO3	1116640.2	68.2	
13.3518	Guanosine	118-00-3	C10H13N5O5	28972901.0	75.6	
13.4224	Dodecahydrocarbazole	6326-88-1	C12H21N	21220020.3	76.4	
13.6534	Dodecahydrocarbazole	6326-88-1	C12H21N	10025992.5	78.4	
13.8202	2,3,7-Trimethylindole	27505-78-8	C11H13N	1234482.1	71.0	
13.8651	9-Azabicyclo[3.3.1]nonan-2-ol, 9-methyl-, acetate (ester), endo-	49656-53-3	C11H19NO2	2211851.5	67.0	
14.0832	Phenol, 2,4-bis(1,1-dimethylethyl)-	96-76-4	C14H22O	7443975.2	93.5	
14.5002	Parbenate	10287-53-3	C11H15NO2	796525.8	66.2	
14.5708	1-Hydroxycyclohexanecarboxylic acid	1123-28-0	C7H12O3	2003328.5	66.5	
14.7183	4-Ethoxy-2-(methylamino)tropone	2000142-04-6	C10H13NO2	7411384.2	84.6	
14.8851	1,5-Naphthyridin-2-amine	17965-80-9	C8H7N3	3223449.5	75.4	
14.9557	6-Ethoxy-1,2,3,4-tetrahydro-2,2,4-trimethylquinoline	16489-90-0	C14H21NO	2319702.8	69.4	
15.1931	1-Hexadecanol, 2-methyl-	2490-48-4	C17H36O	4582144.2	79.5	
15.3214	Pyrrolo[3,2,1-hi]indole, 1,2-dihydro-4,5-dimethyl-	31401-55-5	C12H13N	5037085.7	63.4	
15.4497	Pyrrolo[3,2,1-hi]indole, 1,2-dihydro-4,5-dimethyl-	31401-55-5	C12H13N	14264100.0	82.4	
15.5780	2(3H)-Furanone, dihydro-5-(2-octenyl)-, (Z)-	18679-18-0	C12H20O2	8651371.6	69.2	
15.8474	aR-Turmerone	532-65-0	C15H20O	6382674.6	83.5	
16.4697	1H-Indole-3-acetamide, 2-methyl-.alpha.-oxo-N-(5,6,7,8-tetrahydro-1-naphthalenyl)-	2000628-74-5	C21H20N2O2	30396910.8	79.0	
16.6429	Pentadecanal-	2765-11-9	C15H30O	972013.3	78.9	
16.7584	(E)-4-(3-Hydroxyprop-1-en-1-yl)-2-methoxyphenol	32811-40-8	C10H12O3	762702.9	72.9	
17.5219	Cyclo(L-prolyl-L-valine)	2854-40-2	C10H16N2O2	4800103.2	87.3	
17.6245	2-METHOXY-4-(METHOXYMETHYL)-6-METHYLNICOTINAMIDE	2000234-03-8	C10H14N2O3	4984073.0	64.8	

Component RT	Compound Name	CAS#	Formula	Component Area	Match Factor	Estimated Conc.
17.7271	6-Tetradecanone	6836-42-6	C14H28O	1521232.3	65.4	
18.0287	4-Methylbenzylamine, N-tert-butyldimethylsilyl-	1000417-72-5	C14H25NSi	2944780.4	66.9	
18.2083	N-Propyl-N'-butyl-1,4-benzenediamine	2000223-37-3	C13H22N2	508370.3	60.5	
18.2725	1-Nonadecene	18435-45-5	C19H38	781019.6	74.5	
18.4521	17-Octadecenal (spectrum disagrees)	56554-86-0	C18H34O	1201464.8	72.8	
18.5483	Curcumenone	100347-96-4	C15H22O2	2136736.2	68.3	
18.6959	3-Oxa-8-azaspiro[5.5]undecane, 2,2-dimethyl-8-(2-propynyl)-	87399-75-5	C14H23NO	15771406.8	68.8	
18.8883	2,9-Diazabicyclo[4.4.0]decane, trans-	13623-82-0	C8H16N2	8801767.8	64.2	
19.1385	Phthalic acid, hex-3-yl isobutyl ester	1000356-95-4	C18H26O4	10526649.3	85.8	
19.5619	Pyrrolo[1,2-a]pyrazine-1,4-dione, hexahydro-3-(2-methylpropyl)-	5654-86-4	C11H18N2O2	8302529.6	72.3	
19.6646	11-Methyl-13-tetradecen-1-ol acetate	2000427-93-3	C17H32O2	29558363.8	67.2	
20.0431	Hexahydropyridine, 1-methyl-4-[4-hydroxy-3-methoxyphenyl]-	94427-44-8	C13H19NO2	401929422.1	69.6	
20.1008	Lidocaine	137-58-6	C14H22N2O	199515324.3	88.6	
20.2805	Pentadecanoic acid, ethyl ester	41114-00-5	C17H34O2	163897897.1	80.4	
20.6461	3,6-DIISOBUTYL-2,5-PIPERAZINEDIONE	1436-27-7	C12H22N2O2	6039922.3	82.8	
20.8001	2,5-Piperazinedione, 3,6-bis(2-methylpropyl)-	1436-27-7	C12H22N2O2	7001795.0	82.3	
21.0632	Hexadecanoic acid, methyl ester	112-39-0	C17H34O2	3586189.5	91.9	
21.4481	Hexahydropyridine, 1-methyl-4-[4-hydroxy-3-methoxyphenyl]-	94427-44-8	C13H19NO2	9158273.9	64.9	
22.2243	n-Hexadecanoic acid	57-10-3	C16H32O2	78499321.4	90.8	
22.3976	Ethyl 2-oxohexadecanoate	2000526-05-0	C18H34O3	2977783.1	68.8	
22.5515	Ethanol, 2-(9,12-octadecadienyloxy)-, (Z,Z)-	17367-08-7	C20H38O2	5099716.9	62.5	
22.7504	Hexadecanoic acid, ethyl ester	628-97-7	C18H36O2	76889358.0	98.5	
23.2316	Docosane	629-97-0	C22H46	982681.1	65.4	
24.1490	Ethanol, 2-(9,12-octadecadienyloxy)-, (Z,Z)-	17367-08-7	C20H38O2	451754.0	66.1	
24.8611	9,12-Octadecadienoic acid (Z,Z)-, methyl ester	112-63-0	C19H34O2	5934255.2	94.1	
24.9765	Heptadecanoic acid, ethyl ester	14010-23-2	C19H38O2	5110226.8	86.2	
25.2652	Styrene, 2-nitro-3'-[4-methylphenyloxy]-	2000382-15-2	C15H13NO3	858836.8	62.3	
25.6822	Octadecanoic acid, methyl ester	112-61-8	C19H38O2	3721851.4	80.7	
26.0800	9,12-Octadecadienoic acid (Z,Z)-	60-33-3	C18H32O2	175868371.2	94.7	
26.3623	Linoleic acid ethyl ester	544-35-4	C20H36O2	154711792.7	96.7	
26.4393	9,12,15-Octadecatrienoic acid, ethyl ester, (Z,Z,Z)-	1191-41-9	C20H34O2	41643930.8	85.8	
26.5162	(E)-9-Octadecenoic acid ethyl ester	6114-18-7	C20H38O2	34062528.1	91.5	
26.6253	Ethyl Oleate	111-62-6	C20H38O2	7090124.4	69.3	
27.1000	Octadecanoic acid, ethyl ester	111-61-5	C20H40O2	25792716.4	92.1	
27.2540	i-Propyl 11,12-methylene-octadecanoate	1000336-77-0	C22H42O2	668171.7	66.7	
28.7360	2,5-Piperazinedione, 3-(2-methylpropyl)-6-(phenylmethyl)-, (3S-cis)-	7280-77-5	C15H20N2O2	9914081.6	74.5	
28.9156	2,5-Piperazinedione, 3-(2-methylpropyl)-6-(phenylmethyl)-, (3S-cis)-	7280-77-5	C15H20N2O2	1826214.0	71.3	
29.0824	2-((8Z,11Z)-Heptadeca-8,11-dien-1-yl)-4,5-dihydrooxazole	220556-75-2	C20H35NO	8199946.8	82.3	
29.5700	Cyclohexadecane, 1,2-diethyl-	2000467-33-1	C20H40	7123225.4	70.2	
29.7496	9-Octadecenamide	3322-62-1	C18H35NO	2225247.8	83.4	
30.2885	cis-Methyl 11-eicosenoate	2390-09-2	C21H40O2	1452665.2	60.4	
30.8146	Eicosanoic acid, ethyl ester	18281-05-5	C22H44O2	2521865.4	84.5	
30.9172	(Z,Z)-9,12-octadeca-dienoic acid, 2,3-dihydroxy-propyl ester	2277-28-3	C21H38O4	470638.2	63.0	
31.0198	L-Alanine, .alpha.-N-methyl-N-benzyl-, methyl ester	1000452-62-1	C12H17NO2	2898909.3	74.9	
31.1738	14-.BETA.-H-PREGNA	2000494-05-9	C21H36	448210.1	60.0	
31.6678	2-(Dimethylamino)ethyl (9Z,12Z)-octadeca-9,12-dienoate	116865-17-9	C22H41NO2	1589624.0	66.1	
32.0206	Hexadecanoic acid, 2-hydroxy-1-(hydroxymethyl)ethyl ester	23470-00-0	C19H38O4	1332852.2	62.0	
32.3992	Hexadecanoic acid, 2-hydroxy-1-(hydroxymethyl)ethyl ester	23470-00-0	C19H38O4	54035795.3	89.1	
32.5339	Benzyldiethyl-(2,6-xylylcarbamoylmethyl)-ammonium benzoate	3734-33-6	C28H34N2O3	12301992.4	81.3	
32.8162	3,3'-Dimethyl-4,4'-biphenylene diisocyanate	91-97-4	C16H12N2O2	1344404.6	72.8	
32.9830	Bis(2-ethylhexyl) phthalate	117-81-7	C24H38O4	678718.8	71.7	
33.1369	Hexadecanoic acid, 1-(hydroxymethyl)-1,2-ethanediyl ester	761-35-3	C35H68O5	2388936.8	64.2	
34.1057	Docosanoic acid, ethyl ester	5908-87-2	C24H48O2	2857497.4	78.2	
35.1257	(Z,Z)-9,12-octadeca-dienoic acid, 2,3-dihydroxy-propyl ester	2277-28-3	C21H38O4	105227316.2	91.9	
35.6261	Octadecanoic acid, 2-hydroxy-1-(hydroxymethyl)ethyl ester	621-61-4	C21H42O4	5083010.5	74.7	
35.7480	(Z,Z)-9,12-octadeca-dienoic acid, 2,3-dihydroxy-propyl ester	2277-28-3	C21H38O4	3094590.5	61.2	
36.4409	13-Docosenamide, (Z)-	112-84-5	C22H43NO	7042715.1	89.3	
36.5563	9,12-Octadecadienoic acid (Z,Z)-, 2-hydroxy-1-(hydroxymethyl)ethyl ester	3443-82-1	C21H38O4	2459951.4	63.2	
36.6782	3-Hydroxy-3',4',5'-trimethoxyflavone	154542-32-2	C18H16O6	1281568.9	62.6	
37.0760	Ethyl tetracosanoate	24634-95-5	C26H52O2	4517103.1	88.0	

Component RT	Compound Name	CAS#	Formula	Component Area	Match Factor	Estimated Conc.
37.5443	2,6,10,14,18,22-Tetracosahexaene, 2,6,10,15,19,23-hexamethyl-	7683-64-9	C30H50	2487624.8	82.6	
37.8073	.alpha.-Tocospiro B	601490-41-9	C29H50O4	817706.2	71.5	
38.1409	.alpha.-Tocospiro B	601490-41-9	C29H50O4	594126.0	66.2	
38.4681	Tetracosanoic acid, 2,9-dimethyl-, methyl ester, [S-(R*,S*)]-	55571-05-6	C27H54O2	2518607.3	73.4	
40.3863	Stigmastan-3,5,22-trien	2000773-74-2	C29H46	528262.4	66.4	
41.2652	Cholest-5-en-3-ol (3.beta.)-	57-88-5	C27H46O	6514452.9	94.0	
41.4064	dl-.alpha.-Tocopherol	10191-41-0	C29H50O2	31579482.7	97.0	
41.8426	(22E,24S)-CRINOSTEROL	2000781-08-9	C28H46O	3658280.3	87.5	
42.5483	22,23-METHYLENE-CHOLESTEROL:3RD DIASTEREOMER	0-00-0	C28H46O	1267452.6	72.4	
42.6766	Campesterol	474-62-4	C28H48O	91925396.6	95.9	
43.1129	Stigmasta-5,22-dien-3-ol, (3.beta.,22E)-	83-48-7	C29H48O	173355689.1	93.4	
43.4850	Campesterol, acetate	1000419-55-2	C30H50O2	1105257.3	62.6	
43.7737	.gamma.-Sitosterol	83-47-6	C29H50O	19726220.3	97.3	
44.0110	Lanost-8-en-3-ol, (3.beta.)-	79-62-9	C30H52O	743274.7	70.1	
44.1586	4-Campestene-3-one	51014-22-3	C28H46O	1577792.3	70.9	
44.3831	9,19-Cyclo-27-norlanostan-25-one, 3-(acetyloxy)-24-methyl-, (3.beta.,24R)-	83110-15-0	C32H52O3	749732.5	62.5	
44.6077	9,19-Cyclolanost-24-en-3-ol, (3.beta.)-	469-38-5	C30H50O	3007869.6	76.1	
44.8643	.alpha.-Tocopherol-.beta.-D-mannoside	1000156-68-2	C35H60O7	1490236.0	79.9	
45.3326	9,19-Cyclolanostan-3-ol, 24-methylene-, (3.beta.)-	1449-09-8	C31H52O	1569077.7	70.9	

Sample Chromatogram

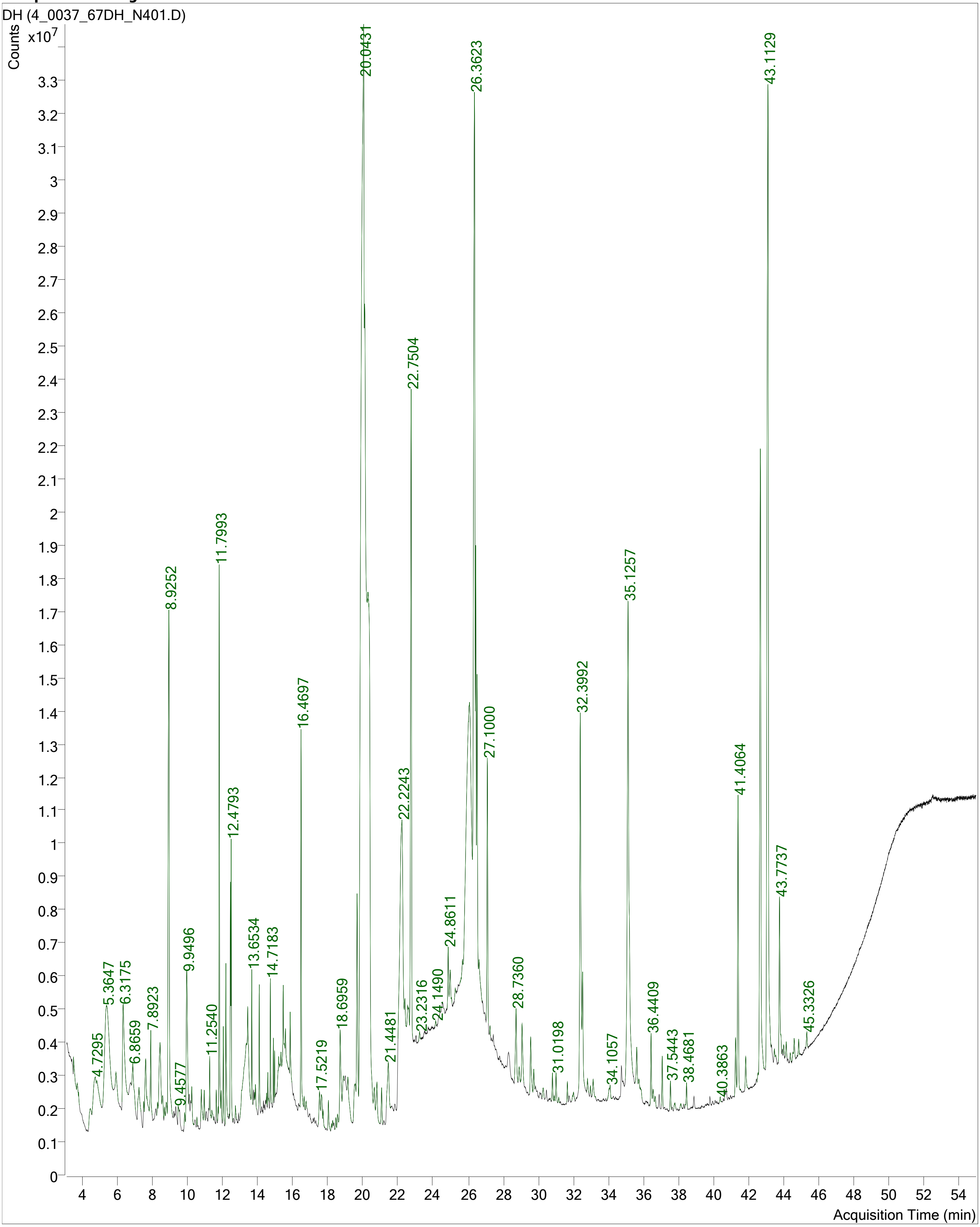

Area Percent Report

Agilent Technologies

Path Name D:\MassHunter\GCMS\1\methods\Method2567\
Acq. Method File 0037_67split1_1M4
Acq. Date-Time 10/19/2023 10:10:52 AM
Sample Name DH
Acq. Method File 0037_67split1_1M4

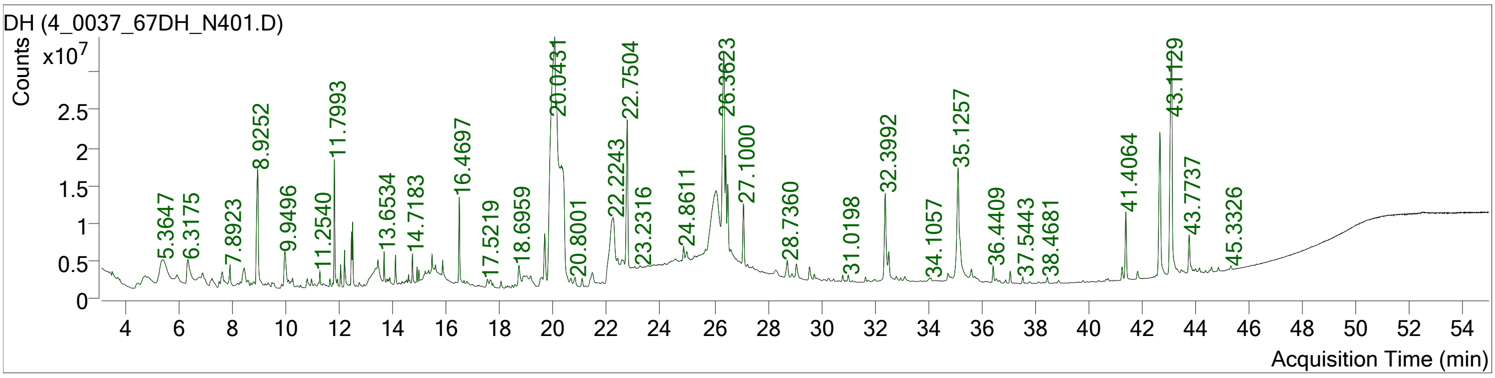

Pk#	RT Min	Start Min	End Min	Peak Height	Peak Area	Peak % Max	% of Total
1	3.2219	3.1963	3.2354	98268.7	151016.6	0.0	0.01
2	3.3823	3.3631	3.4604	177370.2	676462.7	0.2	0.03
3	3.4850	3.4691	3.6453	514925.4	1932696.0	0.5	0.07
4	3.6967	3.6453	3.8189	324399.5	1826203.1	0.5	0.07
5	4.4409	4.3387	4.5307	531871.1	4384867.0	1.1	0.16
6	4.7295	4.5386	4.7809	1056789.4	10606193.1	2.6	0.39
7	4.8129	4.7809	5.0859	810456.2	7708453.3	1.9	0.29
8	5.3647	5.1522	5.7278	2806523.8	48201852.7	12.0	1.78
9	5.9036	5.8458	6.0383	580681.5	3406544.1	0.8	0.13
10	6.3175	6.2439	6.5964	3108972.6	28722700.4	7.1	1.06
11	6.7440	6.5964	6.7953	812225.3	7819791.8	1.9	0.29
12	6.8659	6.7953	7.0265	1391915.8	10578654.0	2.6	0.39
13	7.2187	7.1337	7.3384	732560.6	4407857.0	1.1	0.16
14	7.4818	7.4638	7.5202	345870.9	743169.5	0.2	0.03
15	7.6036	7.5202	7.8025	1650659.0	10673020.6	2.7	0.39
16	7.8923	7.8025	7.9718	2558725.1	7896437.9	2.0	0.29
17	8.1810	8.1179	8.2259	234321.8	851167.3	0.2	0.03
18	8.2773	8.2259	8.3029	441896.5	1228634.7	0.3	0.05
19	8.4248	8.3029	8.5210	2271753.5	16466342.4	4.1	0.61
20	8.5724	8.5210	8.6274	688632.9	3071066.7	0.8	0.11
21	8.6750	8.6437	8.7199	352326.8	911529.2	0.2	0.03
22	8.7777	8.7199	8.8354	520529.2	2428989.1	0.6	0.09
23	8.9252	8.8354	9.1241	15390842.6	74766177.0	18.6	2.77
24	9.2075	9.1690	9.2396	262226.6	748671.9	0.2	0.03
25	9.3166	9.2396	9.4082	462526.2	2427597.3	0.6	0.09
26	9.4577	9.4357	9.5154	420242.6	1560779.8	0.4	0.06
27	9.5411	9.5154	9.5785	399301.3	996563.0	0.2	0.04
28	9.8234	9.7857	9.8747	452920.2	1488346.5	0.4	0.06
29	9.9496	9.8747	10.0543	4673229.3	26291663.3	6.5	0.97
30	10.0864	10.0543	10.1313	984784.5	3522397.2	0.9	0.13
31	10.1698	10.1313	10.1955	788053.7	2611376.2	0.6	0.10
32	10.2275	10.1955	10.3559	1200783.1	4456417.5	1.1	0.16
33	10.4136	10.3559	10.4680	187208.8	727667.5	0.2	0.03
34	10.5227	10.5004	10.5563	249028.0	450983.2	0.1	0.02
35	10.7793	10.7286	10.8883	1064749.6	4652448.7	1.2	0.17
36	10.9397	10.8883	11.0038	1024105.4	3298878.6	0.8	0.12
37	11.0551	11.0038	11.1449	365312.1	1775871.9	0.4	0.07
38	11.1963	11.1449	11.2155	447885.9	1313778.9	0.3	0.05
39	11.2540	11.2155	11.3438	2014362.3	5381094.2	1.3	0.20
40	11.3759	11.3438	11.4850	372286.7	1838166.6	0.5	0.07
41	11.5042	11.4850	11.5483	152868.1	308053.0	0.1	0.01
42	11.6261	11.6009	11.6740	962489.7	2043539.7	0.5	0.08
43	11.7416	11.7080	11.7608	448243.4	814653.5	0.2	0.03
44	11.7993	11.7608	11.8891	16817284.0	38658906.1	9.6	1.43
45	11.9084	11.8891	11.9482	885664.3	1758891.8	0.4	0.07
46	12.0303	11.9798	12.0847	2899775.8	6302685.8	1.6	0.23
47	12.1778	12.1265	12.2933	4866093.5	12866996.5	3.2	0.48
48	12.3639	12.3382	12.3895	283302.8	665987.7	0.2	0.02
49	12.4344	12.3895	12.4601	7255882.0	15472788.5	3.8	0.57

Pk#	RT Min	Start Min	End Min	Peak Height	Peak Area	Peak % Max	% of Total
50	12.4793	12.4601	12.5650	8541005.2	16111739.7	4.0	0.60
51	12.6141	12.5820	12.6526	129489.6	333220.5	0.1	0.01
52	12.7231	12.6984	12.7839	441028.7	1116640.2	0.3	0.04
53	12.9028	12.8835	12.9220	190643.3	251340.4	0.1	0.01
54	13.3518	12.9777	13.3775	2172062.0	28972901.0	7.2	1.07
55	13.4224	13.3775	13.6020	3260177.1	21220020.3	5.3	0.78
56	13.6534	13.6020	13.7239	4318491.6	10025992.5	2.5	0.37
57	13.7624	13.7239	13.7945	662160.8	1888971.3	0.5	0.07
58	13.8202	13.7945	13.8394	569742.0	1234482.1	0.3	0.05
59	13.8651	13.8394	13.9493	798489.0	2211851.5	0.6	0.08
60	14.0832	14.0327	14.1362	3756253.5	7443975.2	1.9	0.28
61	14.2179	14.1923	14.2436	134441.8	245977.1	0.1	0.01
62	14.3270	14.3078	14.3420	182705.1	199273.9	0.0	0.01
63	14.3719	14.3591	14.3976	83608.0	120943.5	0.0	0.00
64	14.4425	14.4253	14.4617	172510.3	238830.5	0.1	0.01
65	14.5002	14.4617	14.5288	373366.4	796525.8	0.2	0.03
66	14.5708	14.5436	14.6345	990384.5	2003328.5	0.5	0.07
67	14.7183	14.6840	14.7795	3783883.8	7411384.2	1.8	0.27
68	14.8851	14.8570	14.9184	1954947.7	3223449.5	0.8	0.12
69	14.9557	14.9319	14.9878	1467272.3	2319702.8	0.6	0.09
70	15.1931	15.1011	15.2444	943757.0	4582144.2	1.1	0.17
71	15.3214	15.2444	15.3599	1007812.7	5037085.7	1.3	0.19
72	15.4497	15.3599	15.5459	2950358.9	14264100.0	3.5	0.53
73	15.5780	15.5459	15.7576	1565955.6	8651371.6	2.2	0.32
74	15.7769	15.7576	15.8089	508421.8	1372972.1	0.3	0.05
75	15.8474	15.8089	15.9520	2180995.9	6382674.6	1.6	0.24
76	16.3286	16.2908	16.4056	186281.8	794652.2	0.2	0.03
77	16.4697	16.4056	16.5916	11470542.3	30396910.8	7.6	1.12
78	16.6429	16.6044	16.6998	394615.2	972013.3	0.2	0.04
79	16.7584	16.7155	16.8247	250130.5	762702.9	0.2	0.03
80	16.9573	16.8803	17.0008	139704.7	533184.6	0.1	0.02
81	17.2203	17.1950	17.2455	112418.5	194234.7	0.0	0.01
82	17.3101	17.2717	17.3436	91012.5	200872.5	0.0	0.01
83	17.5219	17.4466	17.5860	1025506.8	4800103.2	1.2	0.18
84	17.6245	17.5860	17.7015	965720.0	4984073.0	1.2	0.18
85	17.7271	17.7015	17.8399	515093.6	1521232.3	0.4	0.06
86	18.0287	17.9776	18.1485	897319.5	2944780.4	0.7	0.11
87	18.2083	18.1685	18.2404	197120.5	508370.3	0.1	0.02
88	18.2725	18.2404	18.3045	294318.5	781019.6	0.2	0.03
89	18.3302	18.3045	18.3879	225447.5	713743.0	0.2	0.03
90	18.4521	18.3879	18.4970	380473.5	1201464.8	0.3	0.04
91	18.5483	18.4970	18.6125	484807.5	2136736.2	0.5	0.08
92	18.6959	18.6125	18.8049	2888922.2	15771406.8	3.9	0.58
93	18.8883	18.8049	18.9332	1388986.8	8801767.8	2.2	0.33
94	18.9910	18.9332	19.0808	1390987.6	10677669.5	2.7	0.39
95	19.1385	19.0808	19.3660	1357371.6	10526649.3	2.6	0.39
96	19.5619	19.4287	19.6004	1172665.1	8302529.6	2.1	0.31
97	19.6646	19.6004	19.7480	6907373.9	29558363.8	7.4	1.09
98	20.0431	19.7480	20.0752	33139372.2	401929422.1	100.0	14.87
99	20.1008	20.0752	20.2484	24710333.3	199515324.3	49.6	7.38
100	20.2805	20.2484	20.5756	16018867.1	163897897.1	40.8	6.06
101	20.6461	20.5756	20.7039	1088510.1	6039922.3	1.5	0.22
102	20.8001	20.7039	20.9531	1242965.5	7001795.0	1.7	0.26
103	21.0632	20.9994	21.1217	1089823.1	3586189.5	0.9	0.13
104	21.1850	21.1530	21.2556	108536.0	376868.1	0.1	0.01
105	21.4481	21.3319	21.5764	1386466.0	9158273.9	2.3	0.34
106	21.8266	21.7688	21.8972	191257.6	763124.3	0.2	0.03
107	22.2243	21.9962	22.3526	6727217.6	78499321.4	19.5	2.90
108	22.3976	22.3526	22.4874	808501.1	2977783.1	0.7	0.11
109	22.5515	22.4874	22.6606	736250.5	5099716.9	1.3	0.19
110	22.7504	22.6606	22.8411	19492431.9	76889358.0	19.1	2.84
111	23.0263	22.9771	23.0840	221430.6	696291.4	0.2	0.03
112	23.2316	23.1738	23.3126	268560.4	982681.1	0.2	0.04
113	23.5202	23.4457	23.6036	133286.4	653433.7	0.2	0.02
114	23.7191	23.6614	23.7897	160203.4	633995.8	0.2	0.02
115	23.9372	23.8410	24.0078	81961.8	392298.6	0.1	0.01
116	24.1490	24.1105	24.1939	167549.3	451754.0	0.1	0.02
117	24.5531	24.3992	24.6550	322004.9	2326502.0	0.6	0.09

Pk#	RT Min	Start Min	End Min	Peak Height	Peak Area	Peak % Max	% of Total
118	24.8611	24.8016	24.9124	1847424.4	5934255.2	1.5	0.22
119	24.9765	24.9124	25.0903	1056338.0	5110226.8	1.3	0.19
120	25.2652	25.2152	25.3220	277163.4	858836.8	0.2	0.03
121	25.6822	25.5211	25.7079	807844.5	3721851.4	0.9	0.14
122	26.0800	25.7079	26.2532	8724968.5	175868371.2	43.8	6.51
123	26.3623	26.2532	26.4072	27243926.9	154711792.7	38.5	5.72
124	26.4393	26.4072	26.4777	13609823.0	41643930.8	10.4	1.54
125	26.5162	26.4777	26.5932	9753810.1	34062528.1	8.5	1.26
126	26.6253	26.5932	26.7924	1180028.9	7090124.4	1.8	0.26
127	27.1000	27.0346	27.1624	7986813.5	25792716.4	6.4	0.95
128	27.2540	27.2155	27.2797	287997.7	668171.7	0.2	0.02
129	27.4401	27.4092	27.4767	217729.6	433853.1	0.1	0.02
130	27.7993	27.7648	27.8372	125322.3	290288.4	0.1	0.01
131	28.1457	28.0944	28.1971	120067.7	544389.2	0.1	0.02
132	28.3125	28.1971	28.4601	654330.5	5613041.6	1.4	0.21
133	28.7360	28.6406	28.8354	2193130.8	9914081.6	2.5	0.37
134	28.9156	28.8529	28.9798	472724.4	1826214.0	0.5	0.07
135	29.0824	28.9926	29.1899	1801377.2	8199946.8	2.0	0.30
136	29.5700	29.5121	29.6983	1695198.3	7123225.4	1.8	0.26
137	29.7496	29.6983	29.8138	697014.5	2225247.8	0.6	0.08
138	29.8522	29.8138	29.9036	164719.5	512055.4	0.1	0.02
139	30.0896	30.0567	30.1933	153965.4	814326.9	0.2	0.03
140	30.2885	30.2215	30.3519	321936.2	1452665.2	0.4	0.05
141	30.4681	30.3848	30.5148	287568.1	890571.5	0.2	0.03
142	30.8146	30.7591	30.8787	843351.6	2521865.4	0.6	0.09
143	30.9172	30.8787	30.9621	177775.0	470638.2	0.1	0.02
144	31.0198	30.9621	31.0987	872132.1	2898909.3	0.7	0.11
145	31.1738	31.1201	31.2251	160949.7	448210.1	0.1	0.02
146	31.6678	31.6222	31.7127	610278.5	1589624.0	0.4	0.06
147	31.7576	31.7201	31.8414	174552.4	622893.8	0.2	0.02
148	32.0206	31.9037	32.0943	252014.4	1332852.2	0.3	0.05
149	32.3992	32.2965	32.4890	11490286.2	54035795.3	13.4	2.00
150	32.5339	32.4890	32.6728	3259678.0	12301992.4	3.1	0.46
151	32.8162	32.7634	32.8846	398526.1	1344404.6	0.3	0.05
152	32.9830	32.9456	33.0208	284695.3	678718.8	0.2	0.03
153	33.1369	33.0676	33.2951	516777.5	2388936.8	0.6	0.09
154	34.1057	33.9453	34.2019	430383.2	2857497.4	0.7	0.11
155	34.2917	34.2019	34.3337	84365.1	333458.7	0.1	0.01
156	34.7472	34.7156	34.9012	630488.2	2618037.5	0.7	0.10
157	35.1257	34.9717	35.4016	14595430.7	105227316.2	26.2	3.89
158	35.6261	35.5267	35.7031	1197183.5	5083010.5	1.3	0.19
159	35.7480	35.7031	35.9372	388198.7	3094590.5	0.8	0.11
160	36.1393	36.1008	36.2927	89195.3	738205.9	0.2	0.03
161	36.4409	36.3767	36.5050	2185289.0	7042715.1	1.8	0.26
162	36.5563	36.5050	36.6269	511820.9	2459951.4	0.6	0.09
163	36.6782	36.6269	36.7370	320147.5	1281568.9	0.3	0.05
164	36.9028	36.8444	36.9467	384217.2	1124342.0	0.3	0.04
165	37.0760	37.0250	37.1319	1552521.1	4517103.1	1.1	0.17
166	37.5443	37.4972	37.6028	863206.4	2487624.8	0.6	0.09
167	37.8073	37.7348	37.8651	176298.8	817706.2	0.2	0.03
168	38.1409	38.0790	38.2051	148796.8	594126.0	0.1	0.02
169	38.3077	38.2691	38.3413	126259.8	283594.9	0.1	0.01
170	38.4681	38.3661	38.5216	764358.1	2518607.3	0.6	0.09
171	38.8915	38.8241	38.9429	328068.4	1091122.0	0.3	0.04
172	39.7961	39.7563	39.8539	241890.5	666001.5	0.2	0.02
173	39.9501	39.9196	39.9886	79483.9	198661.5	0.0	0.01
174	40.0976	40.0463	40.2131	105140.7	468194.3	0.1	0.02
175	40.3863	40.3359	40.4569	166336.1	528262.4	0.1	0.02
176	40.6558	40.5980	40.6943	270188.8	823495.9	0.2	0.03
177	40.7328	40.6943	40.8053	350806.9	1270784.0	0.3	0.05
178	40.9830	40.9393	41.0188	82101.8	200242.3	0.0	0.01
179	41.2652	41.1967	41.3358	1770603.3	6514452.9	1.6	0.24
180	41.4064	41.3358	41.5668	9023580.9	31579482.7	7.9	1.17
181	41.8426	41.7879	41.9709	979059.0	3658280.3	0.9	0.14
182	42.4521	42.3687	42.4842	171970.1	590852.7	0.1	0.02
183	42.5483	42.4842	42.5740	338466.3	1267452.6	0.3	0.05
184	42.6766	42.5740	42.9140	18907036.3	91925396.6	22.9	3.40
185	43.1129	42.9765	43.2925	29620625.2	173355689.1	43.1	6.41

Pk#	RT Min	Start Min	End Min	Peak Height	Peak Area	Peak % Max	% of Total
186	43.4850	43.4470	43.5873	296142.0	1105257.3	0.3	0.04
187	43.7737	43.7068	43.8956	4965647.7	19726220.3	4.9	0.73
188	43.9212	43.9076	43.9473	87181.8	120814.6	0.0	0.00
189	44.0110	43.9782	44.0489	303137.6	743274.7	0.2	0.03
190	44.1586	44.0831	44.2004	500654.6	1577792.3	0.4	0.06
191	44.3831	44.3460	44.4409	262130.7	749732.5	0.2	0.03
192	44.6077	44.4832	44.6826	646353.9	3007869.6	0.7	0.11
193	44.8643	44.8130	44.9413	486862.8	1490236.0	0.4	0.06
194	45.3326	45.2749	45.3946	474521.3	1569077.7	0.4	0.06
195	52.5307	52.4267	52.7136	170573.8	1491964.6	0.4	0.06
